# Uncertain world: How children’s curiosity and intolerance of uncertainty relate to their behaviour and emotion under uncertainty

**DOI:** 10.1177/17470218241252651

**Published:** 2024-05-28

**Authors:** Zoe J Ryan, Helen F Dodd, Lily FitzGibbon

**Affiliations:** 1 School of Psychology and Clinical Language Sciences, University of Reading, Reading, UK; 2 Children and Young People’s Mental Health Research Collaboration, Exeter Medical School, University of Exeter, Exeter, UK; 3 Division of Psychology, Faculty of Natural Sciences, University of Stirling, Stirling, UK

**Keywords:** Intolerance of uncertainty, curiosity, uncertainty, emotion, behaviour, children

## Abstract

Curiosity and intolerance of uncertainty (IU) are both thought to drive information seeking but may have different affective profiles; curiosity is often associated with positive affective responses to uncertainty and improved learning outcomes, whereas IU is associated with negative affective responses and anxiety. Curiosity and IU have not previously been examined together in children but may both play an important role in understanding how children respond to uncertainty. Our research aimed to examine how individual differences in parent-reported curiosity and IU were associated with behavioural and emotional responses to uncertainty. Children aged 8 to 12 (*n* = 133) completed a game in which they were presented with an array of buttons on the screen that, when clicked, played neutral or aversive sounds. Children pressed buttons (information seeking) and rated their emotions and worry under conditions of high and low uncertainty. Facial expressions were also monitored for affective responses. Analyses revealed that children sought more information under high uncertainty than low uncertainty trials and that more curious children reported feeling happier. Contrary to expectations, IU and curiosity were not related to the number of buttons children pressed, nor to their self-reported emotion or worry. However, exploratory analyses suggest that children who are high in IU may engage in more information seeking that reflects checking or safety-seeking than those who are low in IU. In addition, our findings suggest that there may be age-related change in the effects of IU on worry, with IU more strongly related to worry in uncertain situations for older children than younger children.

Uncertainty, a state of imperfect or unknown information, is pervasive in everyday life and leads to complex patterns of behavioural and affective responses. While humans are generally motivated to resolve uncertainty (Kruglanski et al., 2020), they also tend to find uncertainty uncomfortable or aversive (van Lieshout et al., 2021). Despite these general trends, individuals differ in their responses to uncertainty, resulting in positive and negative behavioural, emotional and cognitive responses (Hillen et al., 2017). While some people find uncertainty exciting and motivating, easily interacting with it, others have difficulty coping with uncertainty and experience psychological distress when faced with it (Stewart et al., 2010). These individual differences in dealing with uncertainty are conceptually associated with curiosity and Intolerance of Uncertainty (IU), respectively. These two constructs come from largely siloed areas of psychological research and there is, therefore, a dearth of research considering them together. Curiosity is typically studied with relation to motivation and education and is associated with enhanced learning in the face of uncertainty (Gruber et al., 2014; von Stumm et al., 2011) along with enhanced wellbeing (Kashdan et al., 2004). In contrast, IU is typically studied from a clinical perspective and is associated with elevated anxiety and worry under uncertainty (Dugas et al., 1998, 2001). Having a better understanding of how children respond to uncertainty and how responses are associated with IU and curiosity has relevance to both children’s learning and their mental health. This study uses a novel behavioural task to examine how children’s responses to uncertainty are associated with individual differences in curiosity and IU.

Curiosity is a complex and multifaceted construct (Grossnickle, 2016), and there is still considerable debate over how it is best defined (Kidd & Hayden, 2015). Here, we follow Jirout and Klahr (2012)’s operationalisation of curiosity as a preference for uncertainty that drives exploratory behaviour. Anecdotally, children exhibit curiosity when given a wrapped (and thus uncertain) gift—they ask questions to establish what is inside, shake it, smell it, explore it and open it, to satiate their curiosity. Indeed, studies of children’s exploratory behaviour have demonstrated that from an early age, children explore and seek information through their interactions with their environment and questioning of more knowledgeable adults (Callanan & Oakes, 1992; Frazier et al., 2009). From infancy, children’s attention is often directed toward uncertain or unpredictable events, maximising their opportunities for experiential learning (Kidd et al., 2012; Schulz & Bonawitz, 2007).

There are individual differences in children’s curiosity, with higher levels of curiosity associated with both academic and wellbeing outcomes. Individual differences in curiosity are associated with differences in academic achievement, showing strong predictive validity over time (Gottfried et al., 2016; Shah et al., 2018). Indeed, in one study, primary-aged children who preferred to explore more uncertain environments in a computer game were found to acquire more information and learn more in an inquiry-based learning context than those who preferred the less uncertain environments (van Schijndel et al., 2018). Kashdan et al. (2004) found that, in adults, curiosity is related to positive affect and life satisfaction, and Jovanovic and Brdaric (2012) found the same in adolescents, along with an association between curiosity and a greater sense of purpose in life. Shoshani (2019) found that in 3- to 6-year-olds, parent-reported curiosity was positively related to a host of other positive character traits including creativity and social intelligence, and that a broader factor of “intellectual strengths” was positively related to emotional wellbeing. Thus, it seems plausible that those with greater curiosity may have more positive affective responses to uncertain situations than those with lower trait curiosity.

A more nuanced approach to the affective character of curiosity can also be considered. As well as positive feelings of interest and excitement at the prospect of new knowledge, sometimes curiosity may be associated with frustration or a sense of deprivation as a result of not having information. Litman and Silvia (2006) suggest that there are two types of curiosity, interest type, which is motivated by the desire for new information and is associated with positive feelings about that information, and deprivation type, which is motivated by a lack of information and is associated with unpleasant feelings of deprivation and frustration until the information is gained. This distinction has been identified in both adults and children (Litman, 2008; Piotrowksi et al., 2014). Recent research with adults suggests that the benefits of curiosity may be more strongly associated with interest- than with deprivation-type curiosity (Whitecross & Smithson, 2023). When measuring children’s trait curiosity, the only validated questionnaire measure is the parent-report questionnaire Epistemic Curiosity in Young Children (I/D-YC) (Piotrowksi et al., 2014). This measure has separate scales for interest- and deprivation-type curiosity, and a two-factor model has been found to provide the best fit although the two factors are strongly correlated. To our knowledge, relationships between children’s behavioural and affective responses to uncertain situations and individual differences in trait curiosity (in general or separated into subtypes) have not previously been explored.

Intolerance of Uncertainty (IU) is a trait characterised by finding uncertainty aversive or distressing (Carleton, 2016a). IU is defined as having a “dispositional incapacity to endure the aversive response triggered by the perceived absence of salient, key, or sufficient information, and sustained by the associated perception of uncertainty” (Carleton, 2016b, p. 32). Despite uncertainty itself being aversive, the perception or presence of threat may bring about a negative response to uncertainty (Osmanağaoğlu et al., 2018; Tanovic et al., 2018). The distress felt by individuals high in IU is thought to be underpinned by dysfunctional processing of uncertainty (Grupe & Nitschke, 2011). It is theorised that, in high IU individuals, uncertainty stimulates worry and that those high in IU are, in turn, more likely to engage with that worrying (Koerner & Dugas, 2008) and have IU “running in the background” as they navigate the world (Hebert & Dugas, 2019).

For people high in IU, feeling uncertain may lead to information seeking behaviour such as compulsive checking of light switches or locks or seeking health related tests and screenings (Fourtounas & Thomas, 2016; Rosen & Knäuper, 2009). Research has shown that adults with Generalised Anxiety Disorder (GAD), a condition strongly associated with IU, use these so-called “safety behaviours” to reduce the discomfort associated with uncertainty (Hebert & Dugas, 2019). For anxious adults, the desire to resolve uncertainty can be so strong that information is sought even when it comes at a cost (Bennett et al., 2020). Thus, while information seeking behaviour may be expected in both those high in IU and highly curious individuals, the affective responses to uncertain situations would be expected to differ between these groups. It seems somewhat self-evident that those who are high in IU would be expected to experience negative affective responses to uncertain situations.

The majority of research examining IU has focused on adults but there is an emerging literature exploring IU in children. This work was reviewed by Osmanağaoğlu et al. (2018) who found that IU has a consistent strong association with both anxiety and worry. The review also highlighted that research has relied almost entirely on questionnaire measures. In the first study to examine behaviour in response to uncertainty and IU in preadolescent children, Osmanağaoğlu et al. (2021) used an adaptation of the Beads task (Jacoby et al., 2014). In this task, children were asked to select beads one at a time from a hidden jar and asked to decide which jar (from a variety of options) the beads were coming from. The colour of the beads in the jars varied at different ratios to provide different levels of uncertainty. On average, as uncertainty increased in the task, information seeking increased, as well as self-reported worry. In relation to IU, Osmanağaoğlu et al. (2021) found that task-related worry was associated with children’s self-reported IU but that neither parent nor child self-reported IU were associated with information seeking. Osmanağaoğlu et al. (2021) interpreted these findings as indicating that when preadolescent children self-report using IU questionnaires, they “capture subjective, affective reactions to uncertainty” (Osmanağaoğlu et al., 2021, p. 7), hence why scores were only associated with self-reported worry and not information seeking behaviour (Osmanağaoğlu et al., 2021; see also, Krain et al., 2006, 2008). Osmanağaoğlu et al. (2021) also theorised that parents may respond to IU questionnaires based on their child’s observable behaviour so we may be more likely to find associations between child behaviour and parent-reported IU, as opposed to child self-report.

Despite the fact that both curiosity and IU describe people’s dispositional responses to uncertainty, to date there is very little empirical or theoretical work bringing these constructs together, largely resulting from these constructs being developed and investigated in siloed research fields. An exception to this is a recent series of studies examining individual differences in adults’ information seeking (Jach et al., 2022; Jach & Smillie, 2021). In these studies, information seeking was assessed for two types of information—information relating to the veracity of arbitrary choices, and information relating to upcoming reward outcomes (whether they had won a bonus in the last game). Curiosity and IU, as well as several other personality traits, were measured via self-report questionnaires with adults. Across these studies, curiosity was more likely to be related to seeking of arbitrary information whereas IU was related to seeking of information about reward outcomes. Interestingly, across these studies, moderate significant positive associations between IU and deprivation-type curiosity and moderate negative associations between IU and interest-type curiosity were found. These authors also found some moderate relationships with other constructs relating to uncertainty such as ambiguity tolerance and openness to experience.

Experimental research has demonstrated that, in general, humans tend to be motivated to resolve uncertainty. Several studies have shown that uncertainty leads to information seeking, even when there is a possibility of a negative outcome (FitzGibbon et al., 2021; Hsee & Ruan, 2016), although these studies are so far limited to adult populations. For example, Hsee and Ruan (2016) explored information seeking in adults by conducting four studies where uncertainty was manipulated and could be resolved by participants but, resolution required participants to risk negative consequences. In their third study (on which the design of the current experiment was based), Hsee and Ruan (2016) presented participants with an array of labelled buttons each indicating that, when pressed, they would play a neutral or aversive sound. There were also buttons labelled with “?” indicating that they could play either the neutral or aversive sound. In the certain condition, there were 44 certain buttons and 4 uncertain, and vice versa for the uncertain condition. Participants were invited to press as many or as few buttons as they liked during the study which would last a few minutes. These authors found that in the uncertain condition participants clicked more buttons than in the certain condition. They also found that the more buttons a participant clicked the worse they felt, and participants in the uncertain condition felt significantly less happy than those in the certain condition. This pattern, which was observed across the series of studies, suggests that the drive to resolve uncertainty can outweigh the negative consequences of doing so. However, individual differences in responses to more or less uncertain situations were not previously explored in these studies, so it is not yet known whether there are individual differences in the motivational underpinnings of their information seeking under uncertainty and the affective responses that correspond with it.

There is currently limited research exploring IU and curiosity together in the context of uncertainty and it is not well understood how IU and curiosity are associated with affect and information seeking behaviour in children. To address this, we created a behavioural task in which uncertainty was manipulated and both affective responses and information seeking behaviour was measured. Our task was based on Hsee and Ruan (2016)’s Study 3 where greater uncertainty was associated with more information seeking, even when there was a possibility of a negative outcome. The task allows information seeking and affect to be captured under varying levels of uncertainty. In case it is not just the aversiveness of the uncertainty causing the response but the presence of possible threat, we have included a mildly aversive stimulus. In the task, children are presented with an array of buttons on the screen that, when clicked, play neutral or aversive sounds. Uncertainty was manipulated between trials by giving the buttons informative (low uncertainty) or uninformative (high uncertainty) labels. Information seeking behaviour was indexed by the number of buttons children pressed. In addition, affective responses were observed via videos of children’s faces and via self-report of emotional valence and worry after initial exposure to each button array.

The first aim of the study was to evaluate whether children’s information seeking is related to curiosity and IU by examining associations between the number of buttons pressed and parent-reported IU and curiosity. We hypothesised that both higher IU and higher curiosity would be associated with more button presses, indicative of greater information seeking. The second aim was to evaluate whether curiosity was associated with positive emotional responses to uncertainty by examining associations between parent-reported curiosity and both the child’s facial affect and self-reported emotional valence. We expected higher curiosity to be associated with positive emotional responses to uncertainty in high uncertainty trials but not in low uncertainty trials. Our final aim was to evaluate whether IU was associated with negative emotional responses to uncertainty, again through the child’s facial affect and self-reported emotional valence, but also through self-reported worry. We hypothesised that higher IU would be associated with more negative emotional responses in higher uncertainty trials.

## Method

### Preregistration

The design, sample size, hypotheses and analysis plan for the study were preregistered on OSF. Further details can be found here.

#### Participants

Participants were 133 children, recruited via two lab databases and social media posts. An a priori power calculation determined that a sample size of 132 participants would be sufficient to reach 80% power for a small interaction effect (standardised beta = 0.02–0.05) based on the variance in pilot data (see the preregistration for further details). Of the 133 participants, 68 were boys, 64 girls and one preferred not to say. Their ages ranged from 8 to 12.96 years (*M* = 9.71 years, *SD* = 1.30). The majority identified as White (111) and 11 as Asian, 2 as Black, 8 as Mixed Race and 1 as Arab. The majority of parents reported completion of a higher education degree (49 Bachelor’s degree, 34 Master’s degree, 33 Postgraduate degree). Of the other parents, three achieved GCSEs, three A Levels, 10 College Course Certificates, and 1 preferred not to say. All children in the study met the following inclusion criteria: they had normal or corrected hearing and vision, were typically developing and were living in the United Kingdom (so that safeguarding procedures relating to video recordings could be followed). This project was approved by the School of Psychology and Clinical Language Sciences Ethics Committee at the University of Reading (2020-072-HD). Further demographic information is available in Supplementary Materials (Table S1).

#### Exclusions

24 additional parents completed the questionnaires but did not meet the above inclusion criteria, so their children were not invited to complete the game. A further 12 questionnaire responses were flagged as suspicious based on their responses to address and name open-text questions– subsequent emails for clarification of responses were ignored therefore these families were not invited to complete the game. Further detail can be found in the Supplementary Materials.

An additional 36 participants were eligible for the study but were not included in the final dataset because their parents did not complete the questionnaires (7), they did not start the game (22), they had technical issues with the game (6), or they withdrew after the practice round (1). Videos recorded from the webcam during the task were checked for parental interference and no participants were excluded on this basis. No participants were excluded after completing the task.

### Parent-report measures

Parents completed questionnaires about their child’s curiosity and IU and provided demographic information via an online form.

#### Curiosity

Children’s trait curiosity was measured using the 10 item parent-report Interest/Deprivation-Young Children (I/D-YC) scale (Litman, 2005; Piotrowksi et al., 2014), which is currently the only validated questionnaire measure of children’s curiosity. The measure has two scales capturing two dimensions of curiosity (Litman, 2005). The I-type subscale captures intellectual interest in obtaining new knowledge with questions such as “My child has fun learning about new topics or subjects” and the D-type subscale captures the desire to obtain knowledge to reduce information deprivation with questions like “My child is bothered when he or she does not understand something and tries to make sense of it.” The I/D-YC uses a 4-point Likert-type scale where 1 indicates “almost never,” 2 “sometimes,” 3 “often” and 4 “almost always.” A validation study with children aged 3 to 8 revealed that the I/D-YC scale demonstrates satisfactory construct validity and acceptable internal consistency for both the I-type (α = .85) and D-type scale (α = .80), and the two subscales are highly correlated (*r* = .84) (Piotrowksi et al., 2014). We also found the subscales to be correlated *r*(131) = 0.47, *p* < .001 (see Table 1), although to a lesser extent than in previous research. Internal consistency for the whole scale was very good in our sample (α = .82). In line with previous research (Jansen et al., 2021) looking at curiosity generally, we combined the two subscales to produce a single measure of curiosity in our main analyses. In addition, we explored the separate effects of the two subscales.

**Table 1.** Mean values, standard deviations, and correlations with 95% confidence intervals of child age, IU (RULES score), and curiosity (I/D-YC total score; interest-type subscale score; and deprivation-type subscale score).

Variable	M (*SD*)	1	2	3	4
1. Child Age (years)	9.71 (1.30)				
2. IU (RULES total score)	34.89 (9.44)	.02 [−.15, .19]			
3. Curiosity (I/D-YC total score)	28.56 (4.70)	−.10 [−.27, .07]	.11 [−.28, .06]		
4. Interest-type Curiosity (I-type subscale score)	16.39 (2.62)	−.15 [−.31, .02]	−.17 [−.33, .00]	.84*** [.79, .89]	
5. Deprivation-type Curiosity (D-type subscale score)	12.17 (2.86)	−.03 [−.20, .14]	−.03 [−.20, .14]	.87*** [.82, .91]	.47*** [.33, .59]

*Note*. ***p < .001. RULES scores are Winsorised.

#### Intolerance of uncertainty

The child’s responses to uncertainty were captured via the 17-item parent-report Responses to Uncertainty and Low Environmental Structure (RULES) questionnaire (Sanchez et al., 2017). The RULES uses a 5-point Likert-type scale where 1 is “not at all,” 3 is “somewhat,” and 5 is “very much,” asking questions such as “My child has a hard time coping with even minor changes” and “My child seeks reassurance prior to entering an unfamiliar situation.” The RULES scale is validated as a measure of children’s IU, has demonstrated convergent validity and strong internal consistency (α = .93) as a parent-report measure in child samples, including those under the age of 10 years (Sanchez et al., 2017). Internal consistency was excellent in our sample (α = .90).

### Task procedure

After completing the questionnaires, parents were sent a link to the Uncertain World online game if the inclusion criteria were met. Completion of the game was “asynchronous,” with no live interaction with the researcher. The game was built using jsPsych (de Leeuw, 2014) in html and JavaScript and run directly from a web server hosted by the University of Reading. The task materials and programme can be found in the GitHub directory in the preregistration (here). Parents were asked to allow the browser access to the computer’s webcam and microphone and to enter a word that was played through the speakers as a means of checking the computer audio. Parents were then asked to hand control of the computer to their child.

First, children completed a practice phase. A computerised voice provided the child with instructions for the game. Children were taught about how the symbols represented neutral (“ok” hand gesture) and aversive (“thumbs down” hand gesture) sounds as well as how the question mark symbol represented uncertain buttons. Children were required to press buttons and hear example sounds in the practice phase. There was then a check to confirm that the child heard the sounds in the practice, and that they wished to proceed with the game. If they answered yes to both of these checks, then they proceeded to the test trials.

**Figure 1.**
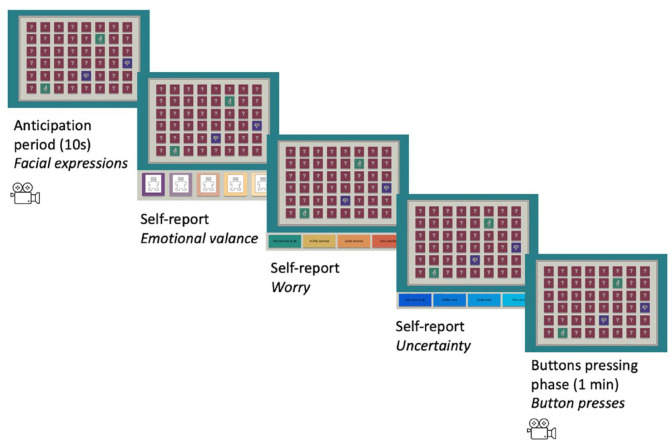
Trial procedure depicting a high uncertainty trial. Self-report measures were displayed until a response was made. Buttons were only responsive in the Button pressing phase. Measures captured during each phase of the trial are shown in italics. Note: Please refer to the online version of the article for the colour version of this figure.

Children completed four test trials, two with high uncertainty, two with low uncertainty (see “Trial design” for more details) in a counterbalanced order. Each of the four test trials consisted of a camera check, an anticipatory period, three self-report questions, and a button pressing phase (see Figure 1). To ensure that the child could be seen by the webcam, they were shown the video feed from the webcam positioned in a spaceship window on the screen. Children were asked to make sure they could see themselves in the window of the spaceship before each trial. When the child was happy, they could see themselves, they clicked a button “Next” to proceed with the trial. In the anticipation period, the 48 buttons for the trial ahead were shown on the screen for 10 s. During this phase, children were instructed to look at the buttons but not press them and their faces were recorded via the webcam for coding of affective responses (see “Facial affect recording, coding, and scoring” section). The child was asked to self-report emotional valence, worry and uncertainty (see “Self-reported emotion valence, worry, and uncertainty” section). In the button pressing phase, the buttons were activated for 1 min, and children could press as many or as few buttons as they liked and hear the sounds. A neutral and an aversive sound was allocated to each of the four trials (see “Trial design” for more details). Neutral and aversive sounds selected from the International Affective Digitised Sounds-2 (IADS-2) database (Bradley & Lang, 2007) were used in the task (see Supplementary Materials for details).

As a manipulation check, once all four trials were completed, the child rated how each sound made them feel. They were then asked to hand over to their parent. The parent was asked to confirm consent for the video to be uploaded, and was then provided with a debrief for the project on screen, with an option to download it. Parents were then sent a £5 voucher as a contribution to their reasonable expenses incurred in taking part in the research.

#### Trial design

**Figure 2.**
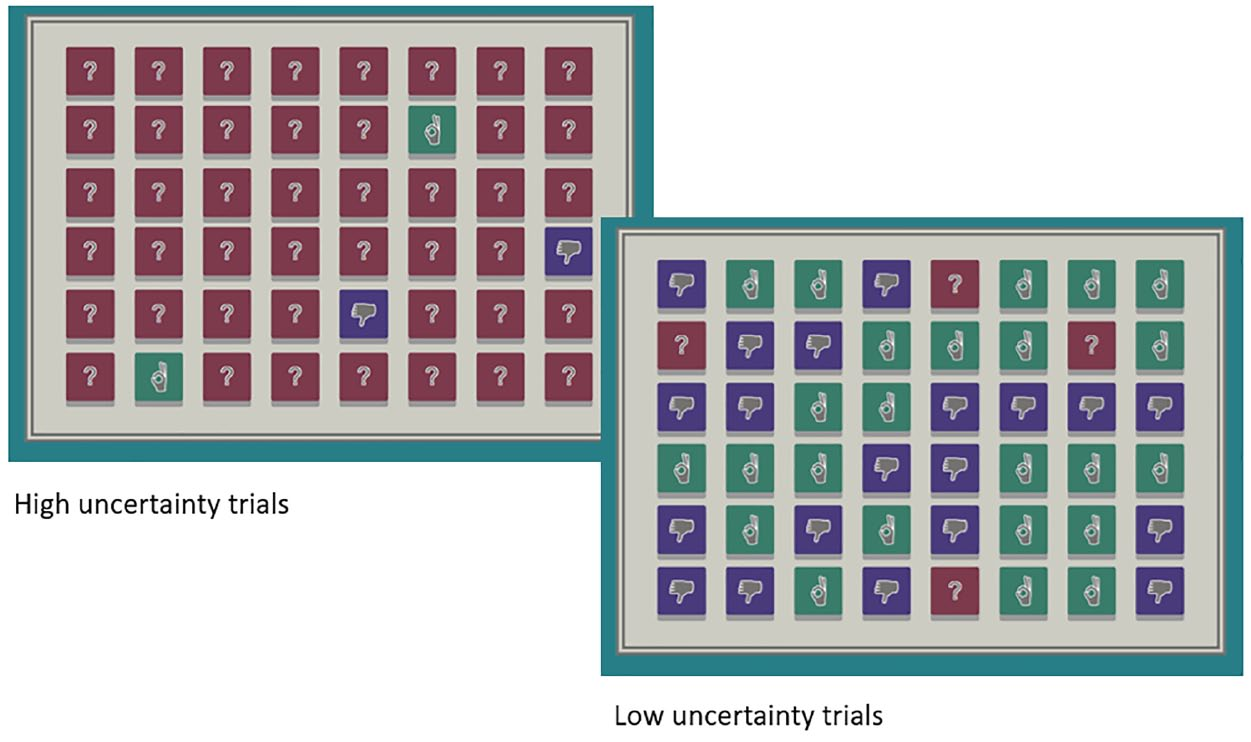
Example button arrays for high and low uncertainty trials. *Note*. Please refer to online version of article for colour version of the figure.

In each of the four trials, 48 buttons were shown on the screen. Allocation of sounds to buttons followed the procedure from Hsee and Ruan (2016)’s buttons task (Experiment 3). The four neutral and four aversive sounds were randomly allocated across the four trials such that one neutral and one aversive sound were allocated to each trial, and those sounds were repeated across relevant buttons in that trial. In high uncertainty trials, 44 buttons were uncertain, and four buttons were certain, two neutral and two aversive. In low uncertainty trials, 4 buttons were uncertain, and 44 buttons were certain, with 22 neutral and 22 aversive (see Figure 2). Where buttons were uncertain, sounds were randomly allocated across the buttons such that half were allocated a neutral sound and half an aversive sound. Buttons were individually mapped onto sounds such that the same sound played each time a specific uncertain button was pressed. When a button was pressed, the sound would play for 2 s during which time the buttons were disabled, resulting in a maximum possible number of button presses of 30 during the 1-min button pressing phase.

### Task measures

#### Button presses

During the button pressing phase, children were invited to press as many or as few buttons as they liked. The number of button presses children made during the button pressing phase on each certain and uncertain trial was recorded. The total number of buttons pressed per trial was used in the analyses (following Hsee and Ruan, 2016). In addition, for exploratory purposes, we calculated the proportion of unique, certain, and uncertain buttons pressed on each trial.

#### Self-reported emotion valence, worry, and uncertainty

To measure children’s emotional valance, we asked children to report how they felt about pressing the buttons in the round on a 5-point Self Assessment Mannikin (SAM; from “very unhappy” to “very happy”) (Bradley & Lang, 1994). Emotional valence rating scores were skewed toward very happy (“very happy” responses were made on 50% of trials).

To measure children’s worry, we asked children to report how worried they felt about the round on a 4-point scale for self-reported worry (from “not at all worried” to “very worried”). Self-report worry rating scores were skewed toward not at all worried (“not at all worried” responses were made on 70% of trials).

As a manipulation check, we measured children’s uncertainty on each trial. We asked children to report how sure or not sure they felt about the sounds they would hear in the round on a 4-point scale from “not at all sure” to “very sure.” This question was arrived upon following piloting and feedback from children that rating uncertainty was more challenging than rating how sure they felt. Responses were therefore reversed to give a rating of uncertainty. Self-report uncertainty rating scores were skewed toward very sure (“very sure” responses were made on 50% of trials).

#### Facial affect recording, coding, and scoring

Children’s faces were recorded during each 10-s anticipation period using the webcam. We preregistered two measures of facial affect, a subjective score and an objective score, as well as a composite of the two. The master coder coded all videos and reliability coder coded 20% of videos as is established practice for determining reliability (see Syed & Nelson, 2015). Coders were blind to the trial uncertainty.

The subjective facial affect score was rated by the coders on a 5-point Likert-type scale per video from –2 (*very unhappy*) to + 2 (*very happy*) based on their overall impression of the child’s affect during the trial. The intra class correlation (ICC) for subjective facial affect score was .68 with a 95% confidence interval from .56 to .77, indicating moderate reliability.

The objective facial affect score was derived as follows. The time spent in smile and frown expressions were coded according to a simple coding scheme developed for this study, based on the Facial Action Coding System (FACS; Ekman & Friesen, 1978) and facial electromyography (fEMG; Cacioppo et al., 1986) methodology. The resulting measure was a proportion score between −1 (*always frowning*) and + 1 (*always smiling*). The ICC for objective facial affect score was .54 with a 95% confidence interval from .39 to .67, indicating poor to moderate reliability. Further detail about the coding and scoring of the objective facial affect score can be found in Supplementary Materials.

The subjective and objective facial affect scores were related to each other, as tested with a multi-level correlation analysis accounting for clustering of trials within participants, *r*(509) = .54, *p* < .001. Given this, we created a composite score by first scaling and then summing the subjective and objective scores. The ICC for the composite score was .69 with a 95% confidence interval from .57 to .78. It was decided that the composite score would be used in the analysis as it had the best ICC from the three facial affect scores, hereafter referred to as the “facial affect score.” Models using subjective and objective facial affect scores are reported in Supplementary Table S7.

#### Self-reported sound ratings

As a manipulation check, we asked children to rate each of the sounds they heard during the task. Each sound was played and children used a 5-point SAM scale (from “very unhappy” to “very happy”) to rate how each sound made them feel.

### Data analysis

#### Missing data and data cleaning

Data on the behavioural task were collected from 133 participants, but video data was missing for four participants due to transfer issues. In addition, video data for three trials was missing for one participant, and video data for one trial was missing from a further two participants. Trials where video data were missing were excluded from facial affect models.

Data for all variables was visualised to detect outliers. Three outliers were identified for RULES and these scores were Winsorised at the participant level (2%), following our preregistered data-analysis plan. Thirteen outliers for the number of button presses (2%) and 11 outliers from the facial affect scores (2%) were Winsorised at the trial level, also following our preregistered data-analysis plan.

Data for all three self-report measures was skewed such that the majority of responses were made at one end of the scale. This skew could not be corrected by transformation. We did not preregister a plan for dealing with skewed data. To assess the robustness of the results with these variables, binary variables were created taking the most frequent response (“Very happy,” “Not at all worried,” and “Very sure”) as one value and any other response as the second value. All planned analyses were repeated using the binarised version of the variable. This did not affect the pattern or significance of the main findings, so the planned analyses are reported and analyses with binary variables are reported in Supplementary Tables S5 and S6. Where differences were noted, these are reported alongside the reported results.

#### Preregistered analysis

The analysis plan was preregistered. The analysis strategy was to assess the predictive value of parent report measures of intolerance of uncertainty (RULES) and curiosity (I/D-YC) on four dependent variables (DVs) capturing children’s behavioural and emotional responses to uncertainty: number of buttons pressed, self-reported emotion, self-reported worry, and facial affect score. The effects of intolerance of uncertainty and curiosity, as well as their interactions with trial uncertainty, were modelled for each dependent variable. Effects of IU and curiosity were first modelled separately (reported in Supplementary Materials Tables S3 S6) and then together, with negligible difference in the model estimates between the approaches.

Linear mixed effects models were run in R (R Core Team, 2022) using the package lme4 (Bates et al., 2014). In all models, random effects for participant (intercepts and random slopes for trial uncertainty) were initially included in the models, but the random effects structure was simplified to intercept only models in all cases to deal with convergence and singular fit errors across all reported models, as described in the preregistration. The syntax for each of the preregistered models reported in this article is as follows:

$$\begin{array}{l} \mathrm{N\_buttons\_pressed}\;\sim \;\left(\mathrm{I/D-YC}\;+\;\mathrm{RULES}\right)\;*\;\mathrm{trial\_uncertainty}\;+\;\left(\;1\;|\;\mathrm{participant}\;\right)\\ \mathrm{sr\_emotion\_valence}\;\sim \;\left(\mathrm{I/D-YC}\;+\;\mathrm{RULES}\right)\;*\;\mathrm{trial\_uncertainty}\;+\;\left(\;1\;|\;\mathrm{participant}\;\right)\\ \mathrm{sr\_worry}\;\sim \;\left(\mathrm{I/D-YC}\;+\;\mathrm{RULES}\right)\;*\;\mathrm{trial\_uncertainty}\;+\;\left(\;1\;|\;\mathrm{participant}\;\right)\\ \mathrm{facial\_affect}\;\sim \;\left(\mathrm{I/D-YC}\;+\;\mathrm{RULES}\right)\;*\;\mathrm{trial\_uncertainty}\;+\;\left(\;1\;|\;\mathrm{participant}\;\right) \end{array}$$

This syntax unpacks to give main effects of trial uncertainty, curiosity, and IU, and interaction effects between trial uncertainty and both curiosity and IU, respectively, but not between the curiosity and IU. Trial uncertainty was effect coded, where low uncertainty was coded −1 and high uncertainty was coded + 1. To make model parameter estimates across RULES and I/D-YC scales comparable, scores for each were converted to z-scores.

#### Additional exploratory analysis

To investigate asymmetry in selection of certain and uncertain buttons across high and low uncertainty trials and whether this could be explained by curiosity and IU, we ran an additional model. On high uncertainty trials, 44 buttons were uncertain and 4 were certain, so certain buttons were in the minority. On low uncertainty trials, 44 buttons were certain and 4 were uncertain, so uncertain buttons were in the minority. To determine whether there was asymmetry in certain and uncertain button pressing that is not related to the different number of each type of button available within a trial, and whether this was associated with trial uncertainty and individual differences in curiosity and IU, we calculated the proportion of minority buttons pressed and used this as the dependent variable in a new model. The syntax for this model is as follows:

$$\begin{array}{c} \mathrm{prop\_minority\_buttons\_pressed}\;\sim \;\left(\mathrm{I/D-YC}\;+\;\mathrm{RULES}\right)\\ {}*\;\mathrm{trial\_uncertainty}\;\\ +\;\left(\;1\;|\;\mathrm{participant}\;\right) \end{array}$$

Given the fairly wide age range included in our study, additional models including age were run to determine whether age moderated the relationships under investigation. The results of these additional analyses are summarised alongside the preregistered analyses and reported in full in Supplementary Table S10. Model syntax follows that of the main analysis with an additional interactive term for age, resulting in two- and three-way interactions between age and trial uncertainty and individual differences measures:

$$\begin{array}{c} \mathrm{dv}\;\sim \left(\mathrm{I/D-YC}\;+\;\mathrm{RULES}\right)\;*\;\mathrm{trial\_uncertainty}\\ \;*\;\mathrm{age}\;+\;\left(\;1\;|\;\mathrm{participant}\;\right) \end{array}$$

To investigate differential effects of Interest- and Deprivation-type curiosity, we repeated the models with each curiosity type. Model syntax follows that of the main analysis but the total scale score was replaced with the subscale scores. The results of these additional models are summarised below and reported in full in the Supplementary Tables S8–S9.

## Results

### Descriptive statistics

Descriptive statistics of child-level factors—children’s age, IU (RULES score) and curiosity (I/D-YC total score, Interest-type subscale score, and Deprivation-type subscale score), as well as their correlations are presented in Table 1. Age was not correlated with IU, curiosity, or either of the curiosity subtypes. IU was not related to curiosity or either of the curiosity subtypes. Both Interest-type and Deprivation-type scores were strongly related to the total I/D-YC score and moderately related to each other.

Descriptive statistics for button presses across high and low uncertainty trials are presented in Table 2, showing the number of buttons pressed, and the proportion of these that were unique, certain, and uncertain buttons. In our analyses, we include the total number of button presses, including instances where children press a button they have pressed before. We note the asymmetry between the proportion of certain and uncertain button presses on high and low uncertainty trials. This is investigated further in additional exploratory analysis, as outlined above.

**Table 2.** Descriptive statistics of button-pressing behaviour.

Trial uncertainty	Number of buttons		Proportion unique		Proportion certain		Proportion uncertain	
High	18.79	(3.30)	0.81	(0.16)	0.31	(0.17)	0.69	(0.17)
Low	18.28	(3.33)	0.84	(0.14)	0.78	(0.12)	0.22	(0.12)

*Note.* Mean (standard deviation) number of buttons pressed per trial (Winsorised) and proportion of unique, certain, and uncertain buttons pressed. One participant did not press any buttons on one low uncertainty trial, and this trial was excluded from the calculation of proportions. Mean values and standard deviations are calculated by first summarising across trials within participants and then across participants to account for clustering.

Descriptive statistics and correlations between affect measures are presented in Table 3. Self-reported emotion valence and worry were negatively related to each other; the happier a child reported feeling, the less worried they reported feeling. Self-reported emotion valence was weakly positively related to the facial affect score; the happier a child reported feeling, the happier the coder rated their facial affect. Self-reported worry was not related to the facial affect score.

**Table 3.** Mean values, standard deviations, and correlations with 95% confidence intervals of affective responses at the trial level.

Variable	*M* (*SD*)	1	2
1. Emotion-valence rating	4.21 (0.74)		
2. Worry rating	1.41 (0.52)	−.48*** [−.55, −.41]	
3. Facial affect score	0.02 (0.63)	.13** [−.04, .22]	−.08 [−.16, .01]

*Note.* Standard deviations are calculated at the participant level. Correlations are adjusted for participant-level clustering.

### Manipulation checks

To check that children recognised that some trials involved more uncertainty than others, the effect of trial uncertainty (high or low) on uncertainty ratings was examined using a linear mixed effect model. There was a small but significant effect of trial uncertainty on uncertainty ratings (*b* = 0.04, 95% CI [0.01, 0.07], *p* = .010), although we note that this was reduced to a trend when self-reported uncertainty was treated as a binary variable (OR = 1.22, 95% CI [0.99, 1.49], *p* = .057). Children reported that they felt less sure about the sounds they would hear on the high uncertainty trials than on low uncertainty trials. See Supplementary Table S2 for full model results.

To check that children found the aversive sounds unpleasant, we conducted a linear mixed effects model on the sound ratings taken at the end of the trial. There was a significant effect of valence on sound ratings (*b* = 0.22, 95% CI [0.18, 0.27], *p* < .001); neutral sounds were rated more positively than aversive sounds. Distributions of rating scores for neutral and aversive sounds are reported in Supplementary Figure S1.

### The influence of uncertainty, IU and curiosity on the number of button presses

**Figure 3.**
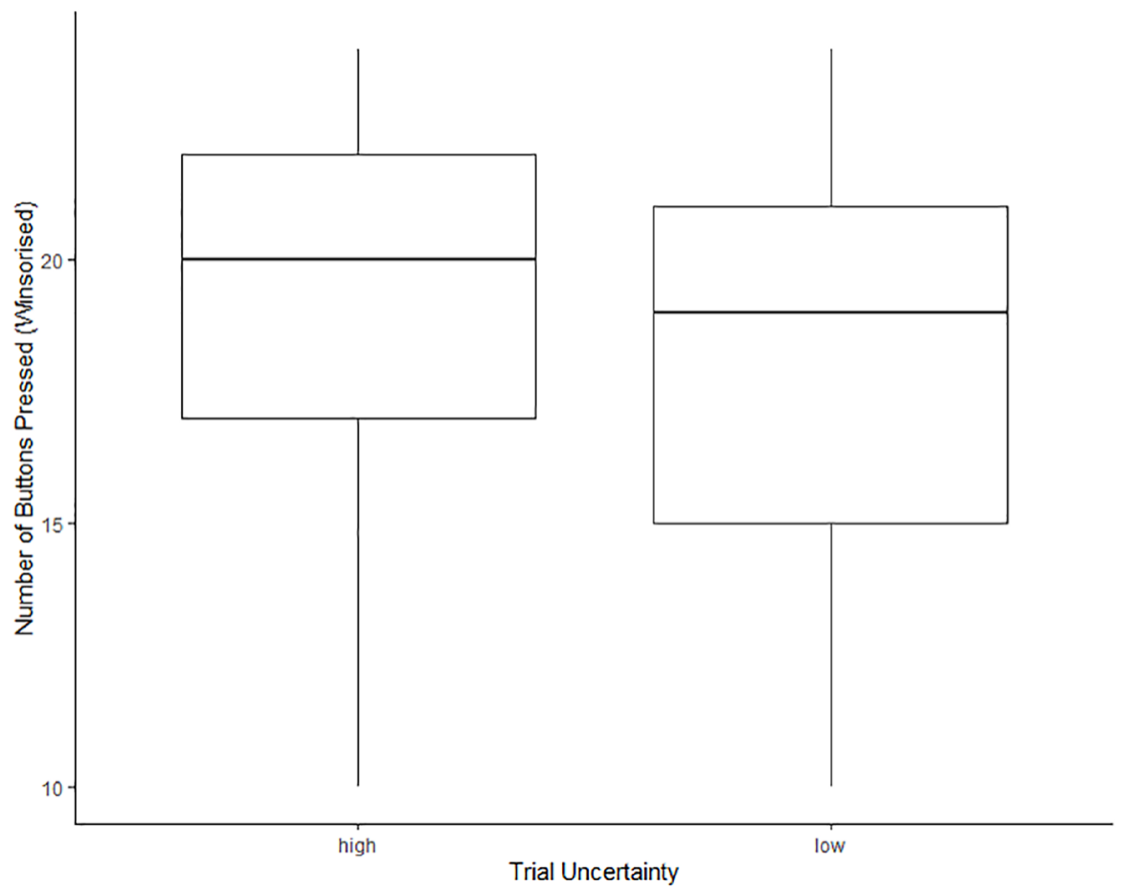
Effect of trial uncertainty on button presses (Winsorised). The lines show the median, whiskers represent scores outside the middle 50% and extend to the minimum and maximum scores, and the box shows the interquartile range (IQR).

We hypothesised that there would be a positive relationship between information seeking under uncertainty (button presses) and both curiosity and IU, reflected in interactions between trial uncertainty and each individual differences measure. Across two separate models (one with IU as a predictor and one with curiosity as a predictor) the following results were found. In both models there was a significant effect of trial uncertainty on buttons presses (*b* = 0.01, 95% CI [0.00, 0.03], *p* = .039). Children pressed significantly more buttons in high uncertainty trials than in low uncertainty trials suggesting that children want to try to resolve uncertainty (see Figure 3), however, this effect was very small, reflected in the mean difference of less than one button in Table 2. IU did not predict button pressing (*b* = −0.02, 95% CI [−0.05, 0.01], *p* = .268). Curiosity did not predict button presses (*b* = 0.01, 95% CI [−0.02, 0.03], *p* = .716). Crucially for our hypotheses, neither IU (*b* = 0.00, 95% CI [−0.01, 0.02], *p* = .509) nor curiosity (*b* = −0.00, 95% CI [−0.02, 0.01], *p* = .554) significantly interacted with trial uncertainty to predict button presses. See Table 4 for LMM results. An exploratory analysis including age did not change the pattern or significance of these effects and no two- or three-way interactions with age were significant, see Supplementary Table S10 for the LMM results.

**Table 4.** LMM results across dependent variables.

Fixed effects												
	Button presses			Facial affect			Self-report emotion valance			Self-report worry		
*Predictors*	*b*	CI	*p*	*b*	CI	*p*	*b*	CI	*p*	*b*	CI	*p*
(Intercept)	**18.53**	**18.01** to **19.05**	**<.001**	0.01	−0.12 to 0.14	.872	**4.21**	**4.09** to **4.33**	**<.001**	**1.41**	**1.32** to **1.50**	**<0.001**
Trial uncertainty	**0.25**	**0.01** to **0.49**	**.039**	*−0.06*	*−0.13* to *0.00*	*.066*	*−0.06*	*−0.13 to 0.00*	*.055*	0.04	*−*0.01 to 0.08	0.145
IU (RULES total)	*−*0.30	*−*0.82 to 0.23	.268	*−*0.05	*−*0.18 to 0.07	.404	*−*0.07	*−*0.19 to 0.05	.266	0.06	*−*0.03 to 0.15	0.176
Curiosity (I/D-YC total)	0.10	*−*0.43 to 0.62	.716	0.06	*−*0.07 to 0.19	.385	**0.20**	**0.07** to **0.32**	**.002**	*−*0.05	*−*0.14 to 0.04	0.269
Trial uncertainty × IU	0.08	*−*0.16 to 0.32	.509	*−*0.02	*−*0.09 to 0.04	.501	0.00	*−*0.06 to 0.07	.895	0.00	*−*0.05 to 0.05	0.953
Trial uncertainty × Curiosity	*−*0.07	*−*0.31 to 0.17	.554	0.05	*−*0.02 to 0.12	.169	*−*0.03	*−*0.09 to 0.04	.385	*−*0.03	*−*0.08 to 0.02	0.262
**Random Effects**												
σ^2^	0.02			0.62			0.55			0.32		
τ_00_	0.02 _id_			0.39 _id_			0.37 _id_			0.19 _id_		
ICC	0.48			0.39			0.4			0.38		
*N*	133 _id_			127 _id_			133 _id_			133 _id_		
Observations	532			503			532			532		
Marginal *R*^2^/Conditional *R*^2^	0.012/0.487			0.014/0.394			0.053/0.432			0.017/0.387		

*Note.* Trial uncertainty is effect coded, RULES total and I/D-YC total are *z*-scored, Button presses and RULES total are Winsorised. Effects significant at the *p* < .05 level are displayed in bold text, trends at *p* < .10 are displayed in italics.

### Exploratory analysis: the influence of uncertainty, IU, and curiosity on type of button pressed

**Figure 4.**
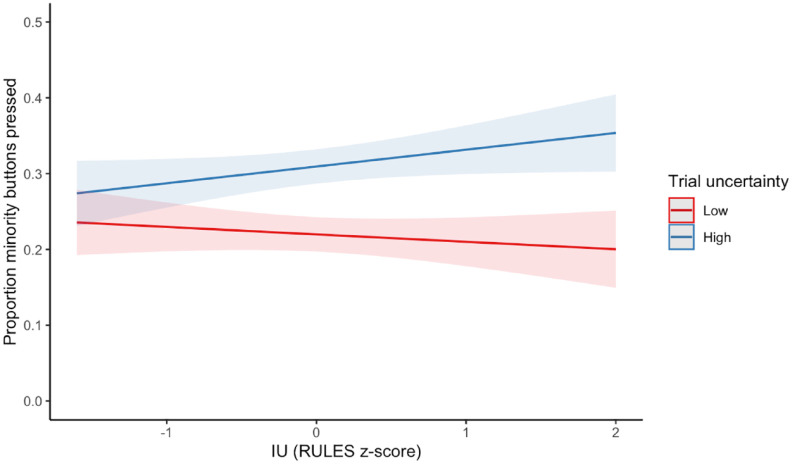
Marginal estimated effects of trial uncertainty and IU on the proportion of minority buttons pressed (certain buttons on high uncertainty trials [blue]/uncertain buttons on low uncertainty trials [red]). Ribbons represent 95% confidence intervals around the estimated effects.

The descriptive statistics suggest that children may have had asymmetrical patterns of pressing the certain and uncertain types of buttons across the high and low uncertainty trials. To investigate this, while accounting for the different numbers of each type of button across trial uncertainties, we calculated the proportion of the minority button pressed and used this as the dependent variable. On high uncertainty trials, the minority buttons are certain (labelled with an “ok” or a “thumbs down” hand gesture) and on low uncertainty trials, the minority buttons are uncertain (labelled with a “?”). There was a main effect of trial uncertainty (*b* = 0.04, 95% CI [0.03, 0.06], *p* < .001), suggesting that children are more likely to press the minority button on high uncertainty trials than on low uncertainty trials. Importantly, this suggests that children press more *certain* buttons on high uncertainty trials than they press *uncertain* buttons on low uncertainty trials. This main effect was qualified by an interaction with IU (*b* = 0.02, 95% CI [0.00, 0.03], *p* = .032). Inspection of the marginal effects suggests that asymmetry in button presses is stronger for those who are high in IU (see Figure 4). No other main effects or interactions were significant. The full model table is available in Supplementary Table S11.

### The influence of uncertainty, IU and curiosity on facial affect

We hypothesised that higher curiosity would be associated with positive facial expressions and that higher IU would be associated with negative facial expressions in response to uncertain situations, reflected in interactions between trial uncertainty and each individual differences measure. There was a trend toward an effect of trial uncertainty on facial affect score (*b* = *−*0.06, 95% CI [*−*0.13, 0.00], *p* = .066). Participants appeared marginally less happy in high uncertainty trials than in low uncertainty trials. IU did not predict facial affect (*b* = *−*0.05, 95% CI [*−*0.18, 0.07], *p* = .404). Curiosity also did not predict facial affect (*b* = 0.06, 95% CI [*−*0.07, 0.19], *p* = .385). Crucially for our hypotheses, neither IU (*b* = *−*0.02, 95% CI [*−*0.09, 0.04], *p* = .501) nor curiosity (*b* = 0.05, 95% CI [*−*0.02, 0.12], *p* = .169) significantly interacted with trial uncertainty to predict facial affect. See Table 4 for LMM results. An exploratory analysis including age did not change the pattern or significance of these effects and no two- or three-way interactions with age were significant, see Supplementary Table S10 for the LMM results.

### The influence of uncertainty, IU and curiosity on self-reported emotional valence

We hypothesised that higher curiosity would be associated with positive self-reported emotional responses and that higher IU would be associated with negative self-reported emotional responses to uncertain situations, reflected in interactions between trial uncertainty and each individual differences measure. There was a trend toward an effect of trial uncertainty on self-reported emotional valence (*b* = *−*0.06, 95% CI [*−*0.13, 0.00], *p* = .055), although this was not robust after the rating score was binarised (see Supplementary Table S5). Participants felt marginally less happy on high uncertainty trials than low uncertainty trials. IU did not predict self-reported emotional valence (*b* = −.07, 95% CI [*−*0.19, 0.05], *p* = .266). There was a significant effect of curiosity on self-reported emotional valence score (*b* = 0.20, 95% CI [0.07, 0.32], *p* = .002). More curious children reporting feeling happier than less curious children. Crucially for our hypotheses, neither IU (*b* = 0.00, 95% CI [*−*0.06, 0.07], *p* = .895) nor curiosity (*b* = *−*0.03, 95% CI [*−*0.09, 0.04], *p* = .385) significantly interacted with trial uncertainty to predict self-reported emotional valance. See Table 4 for LMM results.

**Figure 5.**
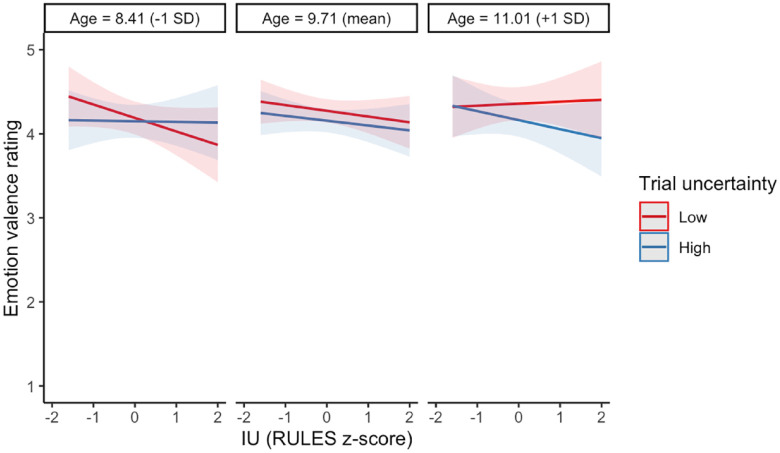
Marginal estimated effects of age, trial uncertainty, and IU on self-reported emotion valence. Ribbons represent 95% confidence intervals around the estimated effects.

An exploratory analysis including age did not change the pattern or significance of these effects. One significant three-way interaction with age was identified between age, trial uncertainty, and IU (*b* = *−*0.05, 95% CI [*−*0.10, *−*0.01], *p* = .030). Visual inspection of the estimated marginal effects suggests that for younger children, higher IU was related to lower happiness on low uncertainty trials, and for older children, higher IU was related to lower happiness on high uncertainty trials (see Figure 5). However, overlapping confidence intervals suggest caution should be taken interpreting this three-way interaction. See Supplementary Table S10 for the LMM results.

### The influence of uncertainty, IU, and curiosity on self-reported worry

We hypothesised that higher IU would be associated with negative self-reported emotional responses to uncertain situations, reflected in an interaction between trial uncertainty and IU. There was no effect of trial uncertainty on self-reported worry (*b* = 0.04, 95% CI [*−*0.01, 0.05], *p* = .145). IU did not predict self-reported worry score (*b* = 0.06, 95% CI [*−*0.03, 0.15], *p* = .176). Curiosity did not predict self-reported worry score (*b* = *−*0.05, 95% CI [–0.14, 0.04], *p* = .269). Crucially for our hypothesis, IU (*b* = –0.00, 95% CI [–0.05, 0.05], *p* = .953) did not interact with trial uncertainty. Curiosity (*b* = –0.03, 95% CI [–0.08, 0.02], *p* = .262) also did not interact with trial uncertainty. See Table 4 for LMM results.

**Figure 6.**
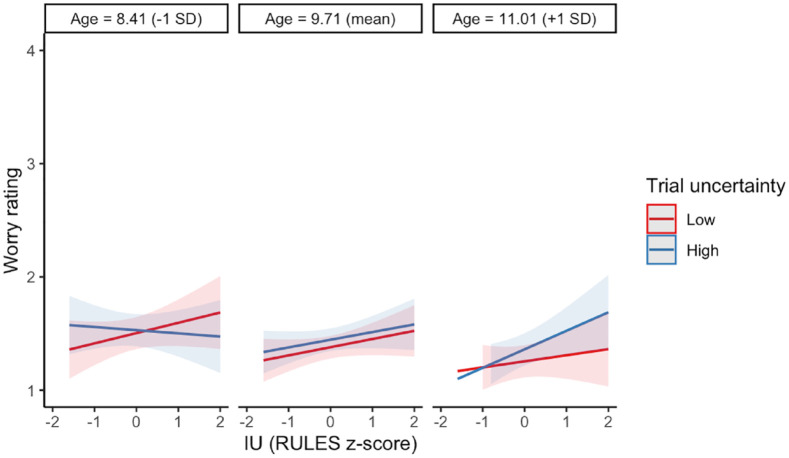
Marginal estimated effects of age, trial uncertainty, and IU on self-reported worry. Ribbons represent 95% confidence intervals around the estimated effects.

An exploratory analysis including age did not change the pattern or significance of these effects. There was a main effect of age such that children became less worried with age (*b* = *−*0.08, 95% CI [*−*0.15, *−*0.01], *p* = .020). A significant three-way interaction with age was identified between age, trial uncertainty, and IU (*b* = 0.04, 95% CI [0.01, 0.08], *p* = .021). Visual inspection of the estimated marginal effects suggests that for younger children, higher IU was related to more worry on low uncertainty trials, and for older children, higher IU was related to more worry on high uncertainty trials (see Figure 6). However, overlapping confidence intervals suggest caution should be taken interpreting this three-way interaction. See Supplementary Table S10 for the LMM results.

### Exploratory analyses: differential effects of interest-type and deprivation-type curiosity

All of the preregistered analyses were replicated, replacing the full I/D-YC scale score with subscale scores for Interest-type and Deprivation-type curiosity respectively. These were modelled separately to avoid issues of multicollinearity. The pattern and significance of the reported effects did not change as a result of including each subscale. Notably, the main effect of curiosity on self-reported emotion valance was seen for both Interest- (*b* = 0.18, 95% CI [0.05, 0.30], *p* = .006) and Deprivation-type (*b* = 0.17, 95% CI [0.04, 0.29], *p* = .009) curiosity. More curious children on both subscales reported feeling happier than less curious children. See Supplementary Tables S8 and S9 for LMM results.

## Discussion

This is the first study to examine IU and curiosity together in a child sample. Furthermore, it is one of the first studies to examine how either IU or curiosity, as separate constructs relate to behaviour and affective responses to uncertainty in children. We hypothesised that children higher in IU and higher in curiosity would engage in more information seeking by pressing more buttons during the task. We also hypothesised that IU would be associated with more negative affect in relation to higher uncertainty and that curiosity would be associated with more positive affect in relation to higher uncertainty. Overall, these hypotheses were not supported; children’s general behaviour under uncertainty in this task was not clearly associated with trait differences in IU or curiosity. However, further exploratory analyses point toward some nuanced effects, especially with respect to IU. The findings are now discussed in relation to each aim.

Our first aim was to evaluate whether children’s information seeking is related to curiosity and IU by examining associations between the number of buttons pressed in the game and parent-reported IU and curiosity. Contrary to our expectations, the number of buttons pressed was not related to IU nor curiosity, however participants overall pressed more buttons in high uncertainty trials than low. Similar to our findings for IU, Osmanağaoğlu et al. (2021) found that preadolescent children’s information seeking behaviour increased as uncertainty increased, but this was not related to IU. We also expected curious children to seek more information to plug the information gap but no effect of curiosity was found. Children tended to press more buttons in higher uncertainty trials irrespective of parent-reported trait curiosity, which suggests that children are generally driven to resolve uncertainty, with little influence of these individual difference variables.

Exploratory investigation of the types of buttons pressed within each trial suggests some more nuanced findings. Although children pressed more buttons in the high uncertainty trials than low uncertainty trials, there was asymmetry in the types of buttons children pressed across the uncertainty conditions. In the high uncertainty trials there were only four certain buttons and in low uncertainty trials there were only four uncertain buttons. Interestingly, children pressed the certain buttons on the high uncertainty trials more often than they pressed the uncertain buttons on low uncertainty trials. Furthermore, this effect was moderated by IU such that the asymmetry was greatest for children high in IU. This increased pressing of the certain buttons in high uncertainty trials could be a checking or safety-seeking behaviour—behaviours that are used to manage the stress of an uncertain or threatening situation (Rachman, 1976; Thwaites & Freeston, 2005). Importantly, this interaction suggests that although IU was not related to quantitative differences in overall button pressing, it was related to qualitative differences in which buttons were pressed. In adults, IU is known to be related to checking and safety-seeking behaviours (Fourtounas & Thomas, 2016; Freeston & Komes, 2023; Hebert & Dugas, 2019), but to our knowledge, this is the first tentative evidence of such a behavioural manifestation of IU in children.

Our second aim was to evaluate whether curiosity was related to positive emotional responses to uncertainty by examining associations between parent-reported curiosity and both the child’s facial affect and self-reported emotional valence during the game. We expected curiosity would be related to positive affect as per Jovanovic and Brdaric (2012) and Kashdan et al. (2004)’s findings in adolescents and adults respectively. We indeed found a small association between curiosity and self-reported positive emotional responses to uncertainty. This effect was robust across both Interest- and Deprivation-type curiosity, suggesting that these subtypes may not have differential associations with affect in childhood. The effect of curiosity on emotion valence was not moderated by level of uncertainty and was not observable in the facial expression data. While this result may support a direct link between curiosity as a trait and positive affect, it is also possible that this link might be driven by the wording of items on the I/D-YC curiosity measure. For example, some questions such as “My child shows visible enjoyment when discovering something new” and “My child has fun learning about new topics or subjects” may capture expression of positive affect. This would explain why curiosity was related to overall happiness, rather than to more positive responses under higher uncertainty. An alternative explanation is that both the high and low uncertainty conditions led to uncertainty-related positive affect because both conditions involve some uncertainty. Having a condition with no uncertainty would have aided interpretation and allowed us to clarify whether the presence of some uncertainty is sufficient to make the more curious children happier or whether they are happier than those low in curiosity even when there is no uncertainty present.

Our final aim was to evaluate whether IU was associated with negative emotional responses to uncertainty. Contrary to our expectations, there were no significant associations or interactions between IU and negative facial affect, self-reported emotional valence or worry. Aligning with our findings about worry, Osmanağaoğlu et al. (2021) found the same lack of worry during a lab-based task in children who were high in parent-reported IU, although they did find associations between child-reported IU and worry. It seems possible given these findings that parent-report IU may not relate to internal states associated with uncertainty, perhaps because they are harder to observe. Thus, it will be important for future research to include child-report measures to further explore this question.

Overall, our findings related to IU were unexpected; for those high in IU, uncertainty elicited neither a negative emotional response, nor increased information seeking. This could be because the parent-report IU questionnaire does not effectively capture associations between IU and affective and behavioural responses to uncertain situations. An alternative explanation is that behavioural and affective responses that are linked to IU are only elicited when sufficient threat is present. It has been theorised that IU is an aversion to uncertainty itself (Carleton, 2016b), however previous research has suggested that the presence of threat may be important (Osmanağaoğlu et al., 2018; Tanovic et al., 2018). Our task may not have included enough threat to stimulate these negative responses. Although participants found the aversive sounds to be relatively negative, they may not have been unpleasant enough to elicit threat-related responses. Future research could address this by introducing a condition with uncertainty but no threat whatsoever, and additional conditions with varying levels of threat (cf. Morriss et al., 2021’s work with adults).

A further alternative explanation is that, given that children could choose whether or not to press buttons during each of the four trials, they may have felt control over their exposure to the sounds. Indeed, our exploratory analyses suggest that children with high IU operated this control by pressing proportionally more certain buttons on uncertain trials. Having this control may have influenced the children’s self-reported worry or affect and may not have induced IU. Future research could address this by manipulating level of control. Control could also be given by allowing children to decide when to end of the trial, rather than having a fixed time interval in which button presses are measured. This would ensure that the number of button presses truly reflects each individuals’ desire for information.

Another possibility is that the relationship between IU and affective responses to uncertainty emerges during middle childhood and would be more prevalent in an older sample. In a series of exploratory analyses, we investigated interactions with age and found that for both self-reported emotion valence and worry, the interaction between trial uncertainty and IU varies by age, with the oldest children in the sample appearing to show the hypothesised pattern of results; IU was related to less happiness and more worry in high uncertainty trials but not in low uncertainty trials. We interpret these exploratory findings with extreme caution because of their exploratory nature and because of overlapping confidence intervals around the marginal estimated effects. We also note that a previous meta-analysis found that age did not moderate the relationship between IU and worry more generally (Osmanağaoğlu et al., 2018). Future research should investigate whether the specific relationship between uncertainty and worry in children with high IU emerges developmentally between the ages of 8 and 12 years, as this may have clinical relevance.

As the first study to examine relationships between curiosity, IU, and behavioural and affective responses to uncertainty in children, this study has several strengths. An important strength is that our manipulation check showed that the task design was successful at manipulating children’s feelings of uncertainty; children reported feeling less sure in high uncertainty trials than low uncertainty trials. This is in line with recent findings suggesting that children’s understanding of terms relating to uncertainty reaches adult levels at around 9 years of age (Meder et al., 2022). Children also sought more information in the uncertain trials, as expected, and their facial affect and self-reported emotion trended toward children feeling less happy in uncertain trials. Furthermore, the aversive sounds were rated more negatively than the neutral sounds. There was also a significant correlation between our measure of facial affect and children’s self-reported emotion valence. Thus, we can be confident that the lack of support for some of our hypotheses does not indicate a design issue with the task; but instead suggests that effects of individual differences in curiosity and IU may not universally affect children’s behavioural or affective responses to uncertainty. A further strength to this study is that it investigates the rarely examined behavioural and affective correlates of IU and curiosity in children. We took an approach that extended the current literature by examining information seeking alongside individual differences in children.

There are however some limitations to the study. One limitation that the uncertainty manipulation check revealed is that while children reported feeling more unsure in the higher uncertainty trials than low, overall they reported feeling quite sure, and the difference between high and low uncertainty trials was small, and was reduced to a trend when the ratings were binarised. Children’s reported lack of uncertainty is particularly surprising since before each trial, children did not know what sounds they were going to hear in either condition. It is possible that children’s interpretation of uncertainty is biased such that they are more confident in the face of uncertainty than adults. Previous research has found that young children tend to be overconfident in relation to their performance in a task (Newman & Wick, 1987; Roebers, 2002) and in the face of uncertainty (Beck et al., 2011; Lapidow et al., 2022). As children have so much uncertainty in their lives as they learn and develop, overconfidence in the face of uncertainty may be an adaptive strategy for coping. The limited number of sounds used in each trial may also have led to a limited sense of uncertainty. Increasing the number of sounds so that each button produces a different sound could increase children’s perceived uncertainty in the task.

A further limitation is that due to the COVID-19 pandemic, the task was run from participants own homes, therefore we could not control the environment in which the child participated, and we could not control the volume of the sounds the buttons made when clicked. Video coding facial affect was particularly challenging, possibly because the design may not be leading to a strong enough emotional response in children. In addition, the inter-rater reliability for the facial affect coding was only modest and facial affect scores were only weakly correlated with children’s self-reported affect and not correlated with self-reported worry, therefore these results need to be interpreted with caution. The children had a relatively flat affect during the anticipation phase; however, recent work argues that facial expressions do not correspond well to emotional states (Barrett et al., 2019) so it may not be a reliable or appropriate method for evaluating affect. Using and refining creative measures to examine affect however should be continued to be explored. For example, Outters et al. (2023) have used body posture change as an indicator of positive affect and task engagement.

Although the online recruitment reduced some of the costs of participating (e.g., the need to travel to the research site), our sample was not very diverse with regard to socioeconomic status and ethnicity. The majority of parents reported having a higher-education degree and were employed either full-time or part-time. This may limit the generalisability of our findings, since both being curious and taking risks may be reliant on the privilege of a safety net if the outcomes do not turn out well; see Giles et al. (2019) and Mabee and Fancher (2020) for further exploration of these ideas with relation to taking risks and curiosity respectively. It is noteworthy that these theoretical ideas have not yet been examined in any detail empirically; this may therefore be an interesting area for future research.

Although there are adult behavioural measures to examine reactions to uncertainty (e.g., Jacoby et al., 2016), there is little convincing evidence yet that IU, as measured by questionnaires, predicts behaviour in children. More generally, a limiting factor in research of this kind is the lack of validated questionnaires that can reliably capture individual differences in children’s responses to uncertainty, making it difficult to tease apart the contributions of different constructs such as IU, curiosity, and other related constructs such as tolerance of ambiguity. Since this study was conducted, a new measure of IU, the Youth Intolerance of Uncertainty—Parent-Report (YIU-PR) (Wong & Caporino, 2023) has been developed which is intended as a developmentally sensitive measure of IU in children and adolescents and may be a more suitable measure of IU in young people going forward. It showed excellent internal consistency and evidenced convergent and discriminant validity. It would be informative to determine whether this new measure is related to Deprivation-type curiosity in children as has been found in adults (Jach et al., 2022; Jach & Smillie, 2021), since we did not replicate this relationship with the RULES as a measure of IU.

Similarly, alternative measures of curiosity that conceptualise curiosity in different ways may reveal different relationships with children’s affective responses to uncertainty. While the I/D-YC was the only validated questionnaire measure of children’s curiosity, we note that the behavioural measure developed by Jirout and Klahr (2012) aligns better with the definition of curiosity as being related to children’s tolerance of uncertainty. In this measure, children’s preferences for uncertainty are calibrated through a series of choices between more or less uncertain stimuli through the task. This measure shows good convergent validity with several other measures of children’s intellectual engagement (e.g., the attitudes toward learning subscale from the Preschool Learning Behaviours Scale, McDermott et al., 2002) and other divergent validity with other cognitive and social factors and so could be a suitable alternative measure of individual differences in children’s curiosity. Further research investigating the relationship between individual differences in this task, and children’s affective responses to uncertainty would be informative about the motivational and affective profiles of children’s curiosity.

In addition, future research would benefit from focusing on children’s thoughts and behaviour in relation to uncertainty and uncertain situations in real life, perhaps using qualitative methods, observational or diary measures. This could inform further experimental work with children. As there is an increasing interest in developing treatments for child anxiety that target reactions to uncertainty specifically, we need a much clearer idea about how responses to uncertainty in children high in IU are distinct to ensure these interventions target the right mechanisms. Understanding curiosity and responses to uncertainty further would also inform educational policies, approaches, and interventions, especially that focus on building motivation for learning.

## Conclusion

IU did not predict children’s emotional responses or the quantity of information seeking during an uncertain task. Exploratory analyses suggest that IU may be associated with the nature of children’s information seeking, with children who are high in IU engaging in more information seeking that reflects checking or safety-seeking than those who are low in IU. In addition, our findings suggest that there may be age-related change in the effects of IU on worry, with IU more strongly related to worry in uncertain situations for older children than younger children. Children higher in curiosity reported feeling happier while completing the task but more uncertainty did not increase happiness in these children. In general, children sought more information under higher uncertainty, but this was not related to curiosity or IU. Future research should focus on establishing how IU manifests in children, monitoring for qualitative as well as quantitative differences in behaviour, and how curiosity can be harnessed to support motivation and learning.

## Supplemental Material

sj-docx-1-qjp-10.1177_17470218241252651 – Supplemental material for Uncertain world: How children’s curiosity and intolerance of uncertainty relate to their behaviour and emotion under uncertainty Supplemental material, sj-docx-1-qjp-10.1177_17470218241252651 for Uncertain world: How children’s curiosity and intolerance of uncertainty relate to their behaviour and emotion under uncertainty by Zoe J Ryan, Helen F Dodd and Lily FitzGibbon in Quarterly Journal of Experimental Psychology
