# Supplementary material for: Uncertain world: How children’s curiosity and intolerance of uncertainty relate to their behaviour and emotion under uncertainty
Source: Q J Exp Psychol (Hove). 2024 May 28;78(4):842–60. doi: 10.1177/17470218241252651 (PMC11905328; doi:10.1177/17470218241252651)
Supplement: sj-docx-1-qjp-10.1177_17470218241252651 – Supplemental material for Uncertain world: How children’s curiosity and intolerance of uncertainty relate to their behaviour and emotion under uncertainty [file sj-docx-1-qjp-10.1177_17470218241252651.docx]

Supplementary Material for:

Uncertain World: How Children’s Curiosity and Intolerance of Uncertainty Relate to their Behaviour and Emotion under Uncertainty.

Zoe J. Ryan^1^, Helen F. Dodd^2,1^ and Lily FitzGibbon^3^

^1^ School of Psychology and Clinical Language Sciences, University of Reading, UK

^2^ Children and Young People’s Mental Health Research Collaboration (ChYMe), Exeter Medical School, University of Exeter.

^3^ Faculty of Natural Sciences, University of Stirling

Correspondence concerning this article should be addressed to Lily FitzGibbon, Faculty of Natural Sciences, University of Stirling, Stirling, FK9 4LA. Email: lily.fitzgibbon@stir.ac.uk

**Supplementary Materials**

## Table of contents

[Supplementary Methods 1](#_Toc152938415)

[Participants 1](#_Toc152938416)

[Exclusions 3](#_Toc152938417)

[Neutral and aversive sounds 3](#_Toc152938418)

[Objective facial affect recording, coding and scoring 3](#_Toc152938419)

[Supplementary results 5](#_Toc152938420)

[Manipulation checks 5](#_Toc152938421)

[Main analyses: Robustness checks 6](#_Toc152938422)

[Exploratory analyses: Differential effects of Interest-type and Deprivation-type curiosity 11](#_Toc152938423)

[Exploratory analyses including age as an interactive predictor 13](#_Toc152938424)

[References 14](#_Toc152938425)

## Supplementary Methods

### Participants

Table S1. Demographic characteristics of full sample

| Characteristic | *N* (%) |
| --- | --- |
| Child gender  Male  Female  Describe their gender differently  Prefer not to say | 68 (51%)  64 (48%)  1 (1%)  0 |
| Child age  8  9  10  11  12 | 47 (35%)  31 (23%)  29 (22%)  19 (14%)  7 (5%) |
| Child ethnicity  White British  White Irish  White European  White Other  Asian or Asian British (Indian origin)  Asian or Asian British (Pakistani origin)  Asian or Asian British (Bangladeshi origin)  Asian or Asian British (Chinese origin)  Asian or Asian British (Other Asian origin)  Black or Black British  Mixed Race  Prefer not to say  Other | 102 (77%)  0  6 (5%)  3 (2%)  8 (6%)  1 (1%)  1 (1%)  0  1 (1%)  2 (2%)  8 (6%)  0  1 (1%) |
| Number of children in household  1  2  3  4 | 23 (17%)  75 (56%)  30 (23%)  5 (4%) |
| Child’s birth order  First born  Second born  Third born  Other | 78 (59%)  38 (29%)  11 (8%)  6 (5%) |
| Child’s handedness  Left-handed  Right-handed | 13 (10%)  120 (90%) |
| Is the child colourblind  Yes  No | 1 (1%)  132 (99%) |
| Respondent’s relationship to child  Mother  Father  Grandmother  Grandfather  Other | 126 (95%)  7 (5%)  0  0  0 |
| Child’s Primary Caregiver?  Yes  No  Shared | 96 (72%)  1 (1%)  36 (27%) |
| Parent age  <30  30-40  41-50  50+ | 0  52 (39%)  73 (55%)  7(1%) |
| Parent marital status  Single  Married  Separated  Divorced  Prefer not to say  Other | 8 (6%)  108 (81%)  1 (1%)  7 (5%)  0  9 (7%) |
| Parent employment status  Employed full-time  Employed part-time  Full-time home-maker  Unemployed  Other | 49 (37%)  51 (38%)  15 (11%)  3 (2%)  15 (11%) |
| Parent level of education  Primary School  GCSEs  A’ Levels  College Course Certificate  Bachelors Degree  Masters Degree  Postgraduate Degree  Prefer not to say | 0  3 (2%)  3 (2%)  10 (8%)  49 (37%)  34 (26%)  33 (25%)  1 (1%) |

### Exclusions

24 parents completed the questionnaires but did not meet the inclusion criteria: nine lived outside of the UK, two did not have a webcam, one child was too old, four children did not have normal or corrected hearing or vision and eight were siblings of children who had already taken part. 12 additional responses were flagged as suspicious: 11 had an address error (i.e. address, city, county, post code not matching) and one had an error with the child’s name (note that addresses were taken as part of the safeguarding procedure for the study).

### Neutral and aversive sounds

Sounds for the game were selected from the International Affective Digitized Sounds-2 (IADS-2) database (Bradley & Lang, 2007). Neutral sounds were shortlisted if they had a medium affective valence rating (quite pleasant) and a medium arousal rating (between calm and excited) and were aversive sounds were shortlisted if they had lower affective valence rating (less pleasant) and higher arousal rating (more excited). These shortlisted sounds were then played to four children within the target age range and they each voted for the four neutral sounds and four sounds they found most aversive. The aversive and neutral sounds with the most votes were chosen for the game. The sounds used as examples in the task were the sound of a rattle (neutral) and an air raid siren (aversive); sounds 134 and 624 of the IADs-2 respectively. In the game itself, the neutral sounds were night, country night, jet and rain; sounds 170, 171, 400 and 627 of the IADS-2 respectively. The aversive sounds used in the game were a rollercoaster, a jackhammer, a buzzer and a dentist drill; sounds 30, 380, 712 and 719 of the IADS-2 respectively. The assignment of the sounds to each trial was randomized between participants so that the order and pairings of the sounds varied.

### Objective facial affect recording, coding and scoring

The FACS Action Unit (AU)12 (lip corner puller) was coded for smiles and AU4 (brow lower) for frown (Ekman et al., 2002; Ekman & Friesen, 1978), in an attempt to mimic facial electromyography (fEMG) recordings for zygomatic major and corrugator supercilii respectively. ELAN software was used for coding of facial expressions in the anticipation period of the videos. Because we were unable to code some portions of the anticipation period due to children covering their face or not facing the camera, time spent smiling and frowning during the anticipation period was converted into two proportion variables and a difference score was calculated to create an objective facial affect score ranging from -1 to 1, where -1 represents the entire codable time spent frowning, and 1 represents the entire codable time spent smiling (*M* = .09, *SD* = .27, range = -1-1). The coders were blind to uncertainty condition. Composite scores were calculated by summing subjective and objective facial affect scores (*M* = .32, *SD* = .79, range = -1.96-2).

## Supplementary results

### Manipulation checks

Table S2. LMM testing effect of trial uncertainty on self-reported uncertainty ratings and repeated using binarised ratings (logistic regression).

| **Fixed Effects** |  | | |  | | |
| --- | --- | --- | --- | --- | --- | --- |
|  | **Uncertainty rating** | | | **Uncertainty rating (Binarised rating)** | | |
| *Predictors* | *b* | *CI* | *p* | *Odds Ratio* | *CI* | *p* |
| (Intercept) | **0.89** | **0.83 – 0.94** | **<.001** | 0.97 | 0.70 – 1.34 | .844 |
| Trial uncertainty | **0.04** | **0.01 – 0.07** | **.010** | *1.22* | *0.99 – 1.49* | *.057* |
| **Random Effects** | |  |  |  |  |  |
| σ^2^ | 0.13 | | | 3.29 | | |
| τ_00_ | 0.08 _id_ | | | 2.19 _id_ | | |
| ICC | 0.38 | | | 0.4 | | |
| N | 133 _id_ | | | 133 _id_ | | |
| Observations | 532 | | | 532 | | |
| Marginal R^2^ / Conditional R^2^ | 0.008 / 0.385 | | | 0.007 / 0.404 | | |

*Note.* Trial uncertainty is effect coded. Effects significant at the *p* < .05 level are displayed in bold text, trends at *p* < .01 are displayed in italics.


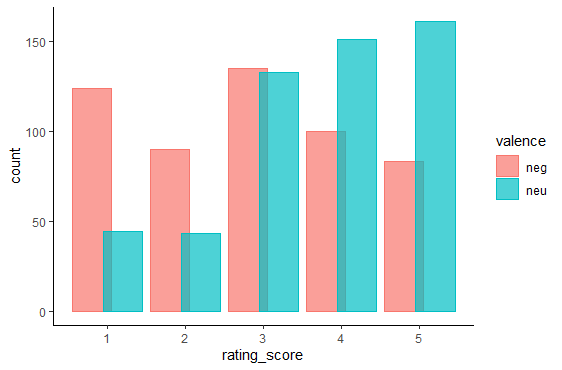


**Figure S1:** Distribution of children’s self-reported emotion valence scores (1 being very unhappy and 5 being very happy) for negative and neutral sounds

### Main analyses: Robustness checks

In our preregistered data analysis plan, we proposed to conduct separate models for IU and Curiosity before combining them in a combined model. There was negligible difference between the model estimates, so only the combined models were presented in the manuscript. Tables of separate and combined model parameters for each dependent variable are presented below (see Tables S3-S6). For models of self-reported emotion valence and self-reported worry, logistic regression models were run with binarised rating scores to test the robustness of the results given the skewed ratings. These are presented alongside the above models for comparison. As an additional robustness check, we present models for the three measures of facial affect for comparison (see Table S7).

Table S3. LMMs for button pressing including IU and Curiosity separately and combined

|  | **Button presses (I/D-YC only)** | | | **Button presses (RULES only)** | | | **Button presses (Combined)** | | |
| --- | --- | --- | --- | --- | --- | --- | --- | --- | --- |
| *Predictors* | *b* | *CI* | *p* | *b* | *CI* | *p* | *b* | *CI* | *p* |
| (Intercept) | 18.53 | 18.01 – 19.05 | **<0.001** | 18.53 | 18.02 – 19.05 | **<0.001** | 18.53 | 18.01 – 19.05 | **<0.001** |
| Trial uncertainty | 0.25 | 0.01 – 0.49 | **0.039** | 0.25 | 0.01 – 0.49 | **0.039** | 0.25 | 0.01 – 0.49 | **0.039** |
| Curiosity (I/D-YC score) | 0.13 | -0.39 – 0.65 | 0.625 |  |  |  | 0.10 | -0.43 – 0.62 | 0.716 |
| Trial uncertainty * Curiosity | -0.08 | -0.32 – 0.16 | 0.503 |  |  |  | -0.07 | -0.31 – 0.17 | 0.554 |
| IU (RULES score) |  |  |  | -0.31 | -0.83 – 0.21 | 0.246 | -0.30 | -0.82 – 0.23 | 0.268 |
| Trial uncertainty * IU |  |  |  | 0.09 | -0.15 – 0.33 | 0.464 | 0.08 | -0.16 – 0.32 | 0.509 |
| **Random Effects** | | | | | | | | | |
| σ^2^ | 7.88 | | | 7.88 | | | 7.89 | | |
| τ_00_ | 7.34 _id_ | | | 7.26 _id_ | | | 7.32 _id_ | | |
| ICC | 0.48 | | | 0.48 | | | 0.48 | | |
| N | 133 _id_ | | | 133 _id_ | | | 133 _id_ | | |
| Observations | 532 | | | 532 | | | 532 | | |
| Marginal R^2^ / Conditional R^2^ | 0.006 / 0.485 | | | 0.011 / 0.485 | | | 0.012 / 0.487 | | |

*Note.* Trial uncertainty is effect coded, RULES total and I/D-YC total are z-scored, Button presses and RULES total are Winsorised. Effects significant at the p < .05 level are displayed in bold text, trends at p < .01 are displayed in italics.

Table S4. LMMs for facial affect including IU and Curiosity separately and combined

|  | **Facial affect (I/D-YC only)** | | | **Facial affect (RULES only)** | | | | **Facial affect (Combined)** | | |
| --- | --- | --- | --- | --- | --- | --- | --- | --- | --- | --- |
| *Predictors* | *b* | *CI* | *p* | *b* | *CI* | *p* | *b* | | *CI* | *p* |
| (Intercept) | 0.01 | -0.12 – 0.14 | 0.877 | 0.01 | -0.12 – 0.14 | 0.877 | 0.01 | | -0.12 – 0.14 | 0.872 |
| Trial uncertainty | *-0.06* | *-0.13 – 0.00* | *0.065* | *-0.07* | *-0.13 – 0.00* | *0.064* | *-0.06* | | *-0.13 – 0.00* | *0.066* |
| Curiosity (I/D-YC score) | 0.06 | -0.07 – 0.19 | 0.341 |  |  |  | 0.06 | | -0.07 – 0.19 | 0.385 |
| Trial uncertainty * Curiosity | 0.05 | -0.02 – 0.12 | 0.148 |  |  |  | 0.05 | | -0.02 – 0.12 | 0.169 |
| IU (RULES score) |  |  |  | -0.06 | -0.19 – 0.07 | 0.356 | -0.05 | | -0.18 – 0.07 | 0.404 |
| Trial uncertainty * IU |  |  |  | -0.03 | -0.10 – 0.04 | 0.422 | -0.02 | | -0.09 – 0.04 | 0.501 |
| **Random Effects** | | | | | | | | | | |
| σ^2^ | 0.62 | | | 0.62 | | | 0.62 | | | |
| τ_00_ | 0.39 _id_ | | | 0.38 _id_ | | | 0.39 _id_ | | | |
| ICC | 0.38 | | | 0.38 | | | 0.39 | | | |
| N | 127 _id_ | | | 127 _id_ | | | 127 _id_ | | | |
| Observations | 503 | | | 503 | | | 503 | | | |
| Marginal R^2^ / Conditional R^2^ | 0.011 / 0.392 | | | 0.009 / 0.389 | | | 0.014 / 0.394 | | | |

*Note.* Trial uncertainty is effect coded, RULES total and I/D-YC total are z-scored, RULES total is Winsorised. Effects significant at the p < .05 level are displayed in bold text, trends at p < .01 are displayed in italics.

Table S5. LMMs for self-reported emotion valence including IU and Curiosity separately and combined and with binarised rating scores

|  | **Emotion valence (I/D-YC only)** | | | **Emotion valence (RULES only)** | | | **Emotion valence (Combined)** | | | **Emotion valence (Combined and binarised rating)** | | |
| --- | --- | --- | --- | --- | --- | --- | --- | --- | --- | --- | --- | --- |
| *Predictors* | *b* | *CI* | *p* | *b* | *CI* | *p* | *b* | *CI* | *p* | *b* | *CI* | *p* |
| (Intercept) | 4.21 | 4.09 – 4.33 | **<0.001** | 4.21 | 4.08 – 4.33 | **<0.001** | 4.21 | 4.09 – 4.33 | **<0.001** | 1.03 | 0.70 – 1.51 | 0.883 |
| Trial uncertainty | -0.06 | -0.13 – 0.00 | 0.055 | -0.06 | -0.13 – 0.00 | 0.055 | -0.06 | -0.13 – 0.00 | 0.055 | 0.84 | 0.67 – 1.05 | 0.119 |
| Curiosity (I/D-YC score) | 0.21 | 0.08 – 0.33 | **0.001** |  |  |  | 0.20 | 0.07 – 0.32 | **0.002** | 1.78 | 1.20 – 2.66 | **0.004** |
| Trial uncertainty * Curiosity | -0.03 | -0.09 – 0.03 | 0.373 |  |  |  | -0.03 | -0.09 – 0.04 | 0.385 | 0.93 | 0.74 – 1.17 | 0.530 |
| IU (RULES score) |  |  |  | -0.09 | -0.22 – 0.03 | 0.155 | -0.07 | -0.19 – 0.05 | 0.266 | 0.91 | 0.62 – 1.34 | 0.632 |
| Trial uncertainty * IU |  |  |  | 0.01 | -0.06 – 0.07 | 0.818 | 0.00 | -0.06 – 0.07 | 0.895 | 0.93 | 0.75 – 1.16 | 0.533 |
| **Random Effects** | | | | | | | | | | | | |
| σ^2^ | 0.55 | | | 0.55 | | | 0.55 | | | 3.29 | | |
| τ_00_ | 0.37 _id_ | | | 0.41 _id_ | | | 0.37 _id_ | | | 3.18 _id_ | | |
| ICC | 0.40 | | | 0.42 | | | 0.40 | | | 0.49 | | |
| N | 133 _id_ | | | 133 _id_ | | | 133 _id_ | | | 133 _id_ | | |
| Observations | 532 | | | 532 | | | 532 | | | 532 | | |
| Marginal R^2^ / Conditional R^2^ | 0.048 / 0.430 | | | 0.013 / 0.430 | | | 0.053 / 0.432 | | | 0.057 / 0.521 | | |

*Note.* Trial uncertainty is effect coded, RULES total and I/D-YC total are z-scored, RULES total is Winsorised. Effects significant at the p < .05 level are displayed in bold text, trends at p < .01 are displayed in italics.

Table S6. LMMs for self-reported worry including IU and Curiosity separately and combined and with binarised rating scores

|  | **Worry (I/D-YC only)** | | | **Worry (RULES only)** | | | **Worry (Combined)** | | | **Worry (Combined and binarised rating)** | | |
| --- | --- | --- | --- | --- | --- | --- | --- | --- | --- | --- | --- | --- |
| *Predictors* | *b* | *CI* | *p* | *b* | *CI* | *p* | *b* | *CI* | *p* | *Odds Ratio* | *CI* | *p* |
| (Intercept) | 0.30 | 0.24 – 0.35 | **<0.001** | 0.30 | 0.24 – 0.35 | **<0.001** | 1.41 | 1.32 – 1.50 | **<0.001** | 0.23 | 0.14 – 0.38 | **<0.001** |
| Trial uncertainty | 0.02 | -0.01 – 0.05 | 0.150 | 0.02 | -0.01 – 0.05 | 0.152 | 0.04 | -0.01 – 0.08 | 0.145 | 1.18 | 0.93 – 1.50 | 0.163 |
| Curiosity (I/D-YC score) | -0.02 | -0.07 – 0.04 | 0.562 |  |  |  | -0.05 | -0.14 – 0.04 | 0.269 | 0.92 | 0.59 – 1.41 | 0.693 |
| Trial uncertainty * Curiosity | -0.03 | -0.06 – 0.00 | 0.079 |  |  |  | -0.03 | -0.08 – 0.02 | 0.262 | 0.80 | 0.63 – 1.03 | 0.086 |
| IU (RULES score) |  |  |  | 0.03 | -0.03 – 0.08 | 0.389 | 0.06 | -0.03 – 0.15 | 0.176 | 1.21 | 0.79 – 1.85 | 0.378 |
| Trial uncertainty * IU |  |  |  | 0.01 | -0.02 – 0.04 | 0.522 | -0.00 | -0.05 – 0.05 | 0.953 | 1.05 | 0.83 – 1.32 | 0.699 |
| **Random Effects** | | | | | | | | | | | | |
| σ^2^ | 0.13 | | | 0.13 | | | 0.32 | | | 3.29 | | |
| τ_00_ | 0.08 _id_ | | | 0.08 _id_ | | | 0.19 _id_ | | | 3.70 _id_ | | |
| ICC | 0.38 | | | 0.37 | | | 0.38 | | | 0.53 | | |
| N | 133 _id_ | | | 133 _id_ | | | 133 _id_ | | | 133 _id_ | | |
| Observations | 532 | | | 532 | | | 532 | | | 532 | | |
| Marginal R^2^ / Conditional R^2^ | 0.007 / 0.381 | | | 0.006 / 0.377 | | | 0.017 / 0.387 | | | 0.018 / 0.538 | | |

*Note.* Trial uncertainty is effect coded, RULES total and I/D-YC total are z-scored, RULES total is Winsorised. Effects significant at the p < .05 level are displayed in bold text, trends at p < .01 are displayed in italics.

Table S7. LMMs for facial affect for comparison of subjective facial affect scores, objective facial affect scores, and composite facial affect scores

|  | **Subjective facial affect** | | | **Objective facial affect** | | | **Composite facial affect** | | |
| --- | --- | --- | --- | --- | --- | --- | --- | --- | --- |
| *Predictors* | *b* | *CI* | *p* | *b* | *CI* | *p* | *b* | *CI* | *p* |
| (Intercept) | 0.35 | 0.23 – 0.47 | **<0.001** | 0.33 | 0.22 – 0.44 | **<0.001** | 0.01 | -0.12 – 0.14 | 0.872 |
| Trial uncertainty | *-0.06* | *-0.12 – 0.00* | *0.062* | -0.04 | -0.11 – 0.03 | 0.264 | *-0.06* | *-0.13 – 0.00* | *0.066* |
| IU (RULES score) | -0.05 | -0.17 – 0.07 | 0.406 | -0.02 | -0.13 – 0.09 | 0.695 | -0.05 | -0.18 – 0.07 | 0.404 |
| Curiosity (I/D-YC score) | 0.08 | -0.04 – 0.20 | 0.211 | 0.04 | -0.07 – 0.15 | 0.483 | 0.06 | -0.07 – 0.19 | 0.385 |
| Trial uncertainty * IU | -0.02 | -0.09 – 0.04 | 0.435 | 0.03 | -0.04 – 0.10 | 0.434 | -0.02 | -0.09 – 0.04 | 0.501 |
| Trial uncertainty * Curiosity | 0.03 | -0.04 – 0.09 | 0.403 | 0.04 | -0.03 – 0.11 | 0.279 | 0.05 | -0.02 – 0.12 | 0.169 |
| **Random Effects** | | | | | | | | | |
| σ^2^ | 0.50 | | | 0.66 | | | 0.62 | | |
| τ_00_ | 0.37 _id_ | | | 0.24 _id_ | | | 0.39 _id_ | | |
| ICC | 0.42 | | | 0.27 | | | 0.39 | | |
| N | 127 _id_ | | | 127 _id_ | | | 127 _id_ | | |
| Observations | 503 | | | 503 | | | 503 | | |
| Marginal R^2^ / Conditional R^2^ | 0.017 / 0.434 | | | 0.007 / 0.275 | | | 0.014 / 0.394 | | |

*Note.* Trial uncertainty is effect coded, RULES total and I/D-YC total are z-scored, RULES total is Winsorised. Effects significant at the p < .05 level are displayed in bold text, trends at p < .01 are displayed in italics.

### Exploratory analyses: Differential effects of Interest-type and Deprivation-type curiosity

Table S8. Models examining dependent variables with Interest (IYC) and Deprivation (DYC) subscales of the I/D-YC separately for button presses and facial affect

|  | **Button Presses (IYC)** | | | **Button Presses (DYC)** | | | **Facial Affect (IYC)** | | | **Facial Affect (DYC)** | | |
| --- | --- | --- | --- | --- | --- | --- | --- | --- | --- | --- | --- | --- |
| *Predictors* | *b* | *CI* | *p* | *b* | *CI* | *p* | *b* | *CI* | *p* | *b* | *CI* | *p* |
| (Intercept) | 18.53 | 18.02 – 19.05 | **<0.001** | 18.53 | 18.01 – 19.05 | **<0.001** | 0.01 | -0.12 – 0.14 | 0.871 | 0.01 | -0.12 – 0.14 | 0.878 |
| Trial uncertainty | 0.25 | 0.01 – 0.49 | **0.039** | 0.25 | 0.01 – 0.49 | **0.039** | *-0.07* | *-0.13 – 0.00* | *0.064* | *-0.06* | *-0.13 – 0.00* | *0.067* |
| IU (RULES score) | -0.27 | -0.80 – 0.25 | 0.308 | -0.31 | -0.83 – 0.21 | 0.247 | -0.05 | -0.17 – 0.08 | 0.489 | -0.06 | -0.19 – 0.07 | 0.359 |
| I-type Curiosity (Interest subscale score) | 0.20 | -0.33 – 0.73 | 0.463 |  |  |  | 0.09 | -0.04 – 0.22 | 0.161 |  |  |  |
| Trial uncertainty * IU | 0.09 | -0.16 – 0.33 | 0.493 | 0.09 | -0.15 – 0.33 | 0.479 | -0.02 | -0.09 – 0.05 | 0.495 | -0.03 | -0.10 – 0.04 | 0.429 |
| Trial uncertainty *  I-type Curiosity | -0.03 | -0.27 – 0.22 | 0.835 |  |  |  | 0.02 | -0.04 – 0.09 | 0.487 |  |  |  |
| D-type Curiosity (Deprivation subscale score) |  |  |  | -0.02 | -0.54 – 0.50 | 0.946 |  |  |  | 0.01 | -0.12 – 0.14 | 0.876 |
| Trial uncertainty * D-type Curiosity |  |  |  | -0.10 | -0.34 – 0.14 | 0.435 |  |  |  | 0.06 | -0.01 – 0.12 | 0.106 |
| **Random Effects** | | | | | | | | | | | | |
| σ^2^ | 7.90 | | | 7.88 | | | 0.62 | | | 0.61 | | |
| τ_00_ | 7.29 _id_ | | | 7.33 _id_ | | | 0.38 _id_ | | | 0.39 _id_ | | |
| ICC | 0.48 | | | 0.48 | | | 0.38 | | | 0.39 | | |
| N | 133 _id_ | | | 133 _id_ | | | 127 _id_ | | | 127 _id_ | | |
| Observations | 532 | | | 532 | | | 503 | | | 503 | | |
| Marginal R^2^ / Conditional R^2^ | 0.013 / 0.487 | | | 0.011 / 0.488 | | | 0.017 / 0.391 | | | 0.012 / 0.395 | | |

*Note.* Trial uncertainty is effect coded, RULES total and I/D-YC subscale scores are z-scored, Button presses RULES total are Winsorised. Effects significant at the p < .05 level are displayed in bold text, trends at p < .01 are displayed in italics.

Table S9. Models examining dependent variables with Interest (IYC) and Deprivation (DYC) subscales of the I/D-YC separately for self-reported emotion valence and worry

|  | **Emotion valence (IYC)** | | | **Emotion valence (DYC)** | | | **Worry (IYC)** | | | | **Worry (DYC)** | | |
| --- | --- | --- | --- | --- | --- | --- | --- | --- | --- | --- | --- | --- | --- |
| *Predictors* | *b* | *CI* | *p* | *b* | *CI* | *p* | *b* | *CI* | *p* | *b* | | *CI* | *p* |
| (Intercept) | 4.21 | 4.09 – 4.33 | **<0.001** | 4.21 | 4.09 – 4.33 | **<0.001** | 1.41 | 1.32 – 1.50 | **<0.001** | 1.41 | | 1.32 – 1.50 | **<0.001** |
| Trial uncertainty | *-0.06* | *-0.13 – 0.00* | *0.055* | *-0.06* | *-0.13 – 0.00* | *0.055* | 0.04 | -0.01 – 0.08 | 0.145 | 0.04 | | -0.01 – 0.08 | 0.145 |
| IU (RULES score) | -0.06 | -0.19 – 0.06 | 0.327 | -0.09 | -0.21 – 0.04 | 0.169 | 0.06 | -0.03 – 0.15 | 0.169 | 0.07 | | -0.02 – 0.15 | 0.148 |
| I-type Curiosity (Interest subscale score) | 0.18 | 0.05 – 0.30 | **0.006** |  |  |  | -0.02 | -0.11 – 0.07 | 0.627 |  | |  |  |
| Trial uncertainty * IU | 0.00 | -0.06 – 0.07 | 0.925 | 0.01 | -0.06 – 0.07 | 0.834 | -0.00 | -0.05 – 0.05 | 0.933 | 0.00 | | -0.05 – 0.05 | 0.972 |
| Trial uncertainty * I-type Curiosity | -0.03 | -0.09 – 0.04 | 0.424 |  |  |  | -0.02 | -0.07 – 0.03 | 0.370 |  | |  |  |
| D-type Curiosity (Deprivation subscale score) |  |  |  | 0.17 | 0.04 – 0.29 | **0.009** |  |  |  | -0.06 | | -0.15 – 0.03 | 0.171 |
| Trial uncertainty * D-type Curiosity |  |  |  | -0.02 | -0.09 – 0.04 | 0.485 |  |  |  | -0.03 | | -0.07 – 0.02 | 0.305 |
| **Random Effects** | | | | | | | | | | | | | |
| σ^2^ | 0.56 | | | 0.56 | | | 0.32 | | | | 0.32 | | |
| τ_00_ | 0.38 _id_ | | | 0.38 _id_ | | | 0.19 _id_ | | | | 0.19 _id_ | | |
| ICC | 0.41 | | | 0.41 | | | 0.38 | | | | 0.37 | | |
| N | 133 _id_ | | | 133 _id_ | | | 133 _id_ | | | | 133 _id_ | | |
| Observations | 532 | | | 532 | | | 532 | | | | 532 | | |
| Marginal R^2^ / Conditional R^2^ | 0.044 / 0.432 | | | 0.041 / 0.432 | | | 0.013 / 0.387 | | | | 0.020 / 0.387 | | |

*Note.* Trial uncertainty is effect coded, RULES total and I/D-YC subscale scores are z-scored, RULES total is Winsorised. Effects significant at the p < .05 level are displayed in bold text, trends at p < .01 are displayed in italics.

### Exploratory analyses including age as an interactive predictor

Table 10. Exploratory LMMs including age as an additional interactive predictor

|  | **Button presses** | | | **Facial Affect** | | | **Emotion valence** | | | **Worry** | | |
| --- | --- | --- | --- | --- | --- | --- | --- | --- | --- | --- | --- | --- |
| *Predictors* | *b* | *CI* | *p* | *b* | *CI* | *p* | *b* | *CI* | *p* | *b* | *CI* | *p* |
| (Intercept) | 18.55 | 18.03 – 19.08 | **<0.001** | 0.01 | -0.10 – 0.13 | 0.820 | 4.21 | 4.09 – 4.34 | **<0.001** | 1.41 | 1.32 – 1.50 | **<0.001** |
| Age | 0.10 | -0.31 – 0.51 | 0.629 | -0.06 | -0.15 – 0.03 | 0.193 | 0.04 | -0.06 – 0.13 | 0.468 | -0.08 | -0.15 – -0.01 | **0.020** |
| Trial uncertainty | 0.24 | 0.00 – 0.48 | **0.049** | *-0.05* | *-0.11 – 0.01* | *0.081* | *-0.06* | *-0.12 – 0.00* | *0.070* | 0.03 | -0.02 – 0.08 | 0.178 |
| IU (RULES score) | -0.28 | -0.82 – 0.25 | 0.296 | -0.04 | -0.16 – 0.07 | 0.437 | -0.06 | -0.19 – 0.06 | 0.322 | 0.07 | -0.02 – 0.16 | 0.121 |
| Curiosity (I/D-YC score) | 0.07 | -0.47 – 0.61 | 0.811 | 0.04 | -0.07 – 0.16 | 0.475 | 0.19 | 0.07 – 0.32 | **0.003** | -0.06 | -0.15 – 0.03 | 0.157 |
| Trial uncertainty * IU | 0.07 | -0.17 – 0.32 | 0.562 | -0.02 | -0.08 – 0.04 | 0.584 | 0.01 | -0.06 – 0.07 | 0.867 | -0.00 | -0.05 – 0.05 | 0.926 |
| Trial uncertainty * Curiosity | -0.05 | -0.30 – 0.20 | 0.702 | 0.04 | -0.02 – 0.10 | 0.168 | -0.04 | -0.11 – 0.02 | 0.187 | -0.02 | -0.07 – 0.03 | 0.517 |
| Age * Trial uncertainty | 0.05 | -0.14 – 0.23 | 0.628 | 0.00 | -0.04 – 0.05 | 0.850 | -0.03 | -0.08 – 0.02 | 0.230 | 0.02 | -0.02 – 0.05 | 0.415 |
| Age * IU (RULES score) | -0.11 | -0.52 – 0.29 | 0.584 | 0.02 | -0.06 – 0.11 | 0.588 | 0.02 | -0.08 – 0.11 | 0.740 | 0.03 | -0.04 – 0.10 | 0.390 |
| Age * Curiosity (I/D-YC score) | 0.12 | -0.30 – 0.54 | 0.565 | 0.02 | -0.07 – 0.11 | 0.687 | 0.05 | -0.05 – 0.15 | 0.321 | 0.04 | -0.03 – 0.11 | 0.220 |
| Age * Trial uncertainty * IU | 0.03 | -0.15 – 0.22 | 0.730 | 0.03 | -0.02 – 0.07 | 0.219 | -0.05 | -0.10 – -0.01 | **0.030** | 0.04 | 0.01 – 0.08 | **0.021** |
| Age * Trial uncertainty * Curiosity | -0.07 | -0.26 – 0.13 | 0.508 | 0.02 | -0.03 – 0.07 | 0.391 | 0.01 | -0.04 – 0.06 | 0.624 | -0.01 | -0.05 – 0.03 | 0.587 |
| **Random Effects** | | | | | | | | | | | | |
| σ^2^ | 7.93 | | | 0.47 | | | 0.55 | | | 0.32 | | |
| τ_00_ | 7.45 _id_ | | | 0.29 _id_ | | | 0.38 _id_ | | | 0.18 _id_ | | |
| ICC | 0.48 | | | 0.39 | | | 0.41 | | | 0.37 | | |
| N | 133 _id_ | | | 127 _id_ | | | 133 _id_ | | | 133 _id_ | | |
| Observations | 532 | | | 503 | | | 532 | | | 532 | | |
| Marginal R^2^ / Conditional R^2^ | 0.017 / 0.493 | | | 0.026 / 0.401 | | | 0.064 / 0.446 | | | 0.055 / 0.402 | | |

*Note.* Trial uncertainty is effect coded, RULES total and I/D-YC subscale scores are z-scored, Button presses RULES total are Winsorised. Effects significant at the p < .05 level are displayed in bold text, trends at p < .01 are displayed in italics.

**Exploratory analysis: The influence of uncertainty, IU, and curiosity on type of button pressed**

Table S11. Exploratory LMM with proportion minority buttons pressed as the dependent variable

|  | **Proportion minority buttons pressed** | | |
| --- | --- | --- | --- |
| *Predictors* | *b* | *CI* | *p* |
| (Intercept) | 0.26 | 0.25 – 0.28 | **<0.001** |
| Trial uncertainty | 0.04 | 0.03 – 0.06 | **<0.001** |
| IU (RULES score) | 0.01 | -0.01 – 0.02 | 0.492 |
| Curiosity (I/D-YC score) | 0.00 | -0.01 – 0.02 | 0.657 |
| Trial uncertainty * IU | 0.02 | 0.00 – 0.03 | **0.032** |
| Trial uncertainty * Curiosity | -0.00 | -0.02 – 0.01 | 0.571 |
| **Random Effects** | | | |
| σ^2^ | 0.03 | | |
| τ_00_ _id_ | 0.00 | | |
| ICC | 0.10 | | |
| N _id_ | 133 | | |
| Observations | 531 | | |
| Marginal R^2^ / Conditional R^2^ | 0.068 / 0.166 | | |

## References

Bradley, M. M., & Lang, P. J. (2007). The International Affective Digitized Sounds (2nd Edition; IADS-2): Affective ratings of sounds and instruction manual. Technical report B-3. *University of Florida, Gainesville, Fl.*

Ekman, P., Friesen, W., & Hager, J. (2002). Facial action coding system [E-book]. *Salt Lake City, UT: Research Nexus*.

Ekman, P., & Friesen, W. V. (1978). Facial action coding system. *Environmental Psychology & Nonverbal Behavior*.
